# Effects of Prolonged Normobaric Hypoxia and Norepinephrine on the Rat Heart—Can They Be Reversed by Normoxic Recovery?

**DOI:** 10.3390/cells15131207

**Published:** 2026-07-02

**Authors:** Beate Rassler, Charly Bambor, Sarah Daunheimer, Coralie Raffort, Aida Salameh

**Affiliations:** 1 Carl-Ludwig-Institute of Physiology, Medical Faculty, Leipzig University, 04103 Leipzig, Germany; charly.bambor@gmx.net (C.B.); sarah.daunheimer@gmx.de (S.D.); 2 Department of Pediatric Cardiology, Heart Centre, Leipzig University, 04289 Leipzig, Germany; coralie.raffort@uni-leipzig.de (C.R.); aida.salameh@medizin.uni-leipzig.de (A.S.)

**Keywords:** hypoxia/reoxygenation, norepinephrine, cardiac function, left and right ventricular catheterization, oxidative/nitrosative stress, nitrotyrosine, PARylation, parthanatos, apoptosis-inducing factor

## Abstract

**Highlights:**

We investigated the reversibility of left ventricular (LV) dysfunction and myocardial cell damage caused by three days of hypoxia and/or norepinephrine (NE) following three days of normoxic recovery.

**What are the main findings?**

- The hypoxia-induced LV functional impairments resolved completely during normoxic recovery after both NaCl and NE infusions.
- Oxidative/nitrosative stress, PARylation, and AIF release partially persisted throughout the reoxygenation phase.

**What are the implications of the main findings?**

- Myocardial cell damage is largely reversible, suggesting that parthanatos probably plays a minor role in hypoxia-induced LV dysfunction during subchronic hypoxia and subsequent reoxygenation.
- Additional NE administration increases oxidative/nitrosative stress in the heart but also causes mainly reversible cellular damage and does not increase parthanatos.

**Abstract:**

Previous studies on rats showed a deterioration of left ventricular (LV) function and myocardial injury characterized by oxidative/nitrosative stress, PARylation, and apoptosis in the heart after three days of hypoxia. In the present study on rats, we investigated whether a three-day recovery period in normoxia can reverse myocardial injury and dysfunction. Further, we studied the effects of norepinephrine (NE) administration as a model of strong sympathetic activation on hypoxia-induced LV dysfunction and myocardial damage, as well as their reversibility. Three days of normobaric hypoxia (10% O_2_) significantly decreased LV systolic function. Contrary to our expectations, NE infusion even aggravated the depression in LV function. These dysfunctions were completely reversed after three days of normoxic recovery. In contrast, nitrotyrosine as a marker of oxidative/nitrosative stress receded incompletely, and poly(ADP-ribose) (PAR) levels were even higher after the recovery period. However, apoptosis-inducing factor receded, at least partially indicating that PAR-related apoptosis (parthanatos) is probably not a major cause of hypoxia-induced LV dysfunction. Additional administration of NE mildly aggravated oxidative/nitrosative stress but did not significantly intensify PARylation and consequently, parthanatos. The findings demonstrate that hypoxia-induced LV dysfunction is reversible, suggesting that subchronic hypoxia and subsequent reoxygenation might have a better prognosis for the LV than classical ischemia/reperfusion injury.

## 1. Introduction

Stays in hypoxic environments, e.g., at high altitudes or in rooms with low oxygen content, challenge all organs and tissues. A low arterial oxygen partial pressure (paO_2_) leads to poor oxygen supply to the tissues and energy depletion, which can ultimately result in reduced organ function. The heart is a high-energy-consuming organ and is absolutely dependent on aerobic energy production. Even minor reductions in oxygen availability lead to changes in heart function, which then—due to impaired circulation—affect other organs, further reducing their oxygen supply. Previous studies on rats showed a significant decrease in cardiac pump function after three days of normobaric hypoxia at 10% O_2_, as indicated by significant reductions in left ventricular systolic peak pressure (LVSP) and left ventricular (LV) contractility, heart rate (HR), stroke volume (SV), and cardiac index (CI) [1]. The decrease in LVSP and LV contractility became significant at as early as after 6 h of hypoxia exposure, while CI remained unaltered during the first 16 h of hypoxia exposure and only began to decline after 24 h [2]. Prolonged exposure to hypoxia over 6 days further deteriorated CI due to a further reduction in HR, despite a recovery of LVSP and LV contractility [3].

In contrast, acute hypoxia in humans is associated with an increase in cardiac output resulting from tachycardia in combination with unchanged stroke volume [4,5,6]. The stroke volume can be maintained by improved LV twist mechanics, as has been demonstrated in echocardiographic studies [6,7,8]. This improvement in cardiac function is considered to result from sympathetic activation, which is stimulated by peripheral chemoreceptors [9,10]. In the first hours of hypoxia, the hypoxia-induced systemic vasodilation tends to override sympathetic vasoconstriction. Consequently, the total peripheral resistance (TPR) decreases, and the blood pressure in the systemic circulation remains largely constant or rises only slightly [10,11,12].

One possible reason for the significant decline in LV systolic function in rats under hypoxia could be that sympathetic activation is weak. This was indicated by insignificant increases in serum norepinephrine (NE) and epinephrine concentrations in rats exposed to hypoxia for up to 24 h. Moreover, additional adrenergic blockade only slightly reduced LV systolic pressure and contractility, HR, and cardiac output compared to hypoxia without adrenergic blockade [2]. After three days of hypoxia, however, β-adrenergic blockade (alone or on combination with α_1_-adrenergic blockade) induced a significant reduction in LV contractility, SV, HR and CI [1]. Studies on humans have shown that sympathetic activation increases considerably with longer exposure to hypoxia [13,14]. It is noteworthy, however, that hypoxia also increases NE clearance [9] and induces downregulation of β-adrenergic receptors [15], which may attenuate the effects of sympathetic activation and thus prevent recovery of LV function.

Another reason for the LV depression in hypoxic rats can be found in the restricted metabolic situation caused by the reduced oxygen supply. Under hypoxic conditions, most cells, including cardiomyocytes, shift their metabolism towards reduced mitochondrial respiration and increased anaerobic glycolysis, resulting in a reduced production of adenosine triphosphate (ATP) [16,17,18]. Further, hypoxia impairs mitochondrial electron transport, leading to increased production of reactive oxygen species (ROS) such as superoxide anions. Several mechanisms are involved in mitochondrial ROS production that are mediated both by hypoxia directly and by hypoxia-inducible factor (HIF) signaling pathways, of which HIF-1 is the most important [19,20]. ATP deficiency exacerbates mitochondrial ROS formation via an increase in intracellular calcium concentration. This calcium increase causes a mitochondrial calcium overload and leads to severe mitochondrial dysfunction, massive exacerbation of energy depletion and ROS production, and finally, cell demise [21,22]. In addition, hypoxia increases the expression of inducible nitric oxide (NO) synthase and consequently, NO synthesis [23]. When excessively produced, NO binds to the superoxide radical, and the highly cytotoxic reactive nitrogen species (RNS) peroxynitrite is formed [24]. Peroxynitrite causes the oxidation and nitration of numerous proteins and lipids. As it is a short-lived and highly reactive oxidant, it is difficult to detect, but nitrotyrosine (NT), the stable product of peroxynitrite-mediated tyrosine nitration, is established as a relevant biomarker of oxidative/nitrosative stress [25]. Besides causing the exacerbation of oxidative damage to mitochondrial proteins and membranes, peroxynitrite induces DNA injuries, including single strand breaks [26]. These DNA injuries activate energy-consuming repair mechanisms, including the ribosylation of poly-adenosine diphosphate (ADP), forming poly(ADP-ribose) (PAR), which is also triggered by peroxynitrite [27]. In cases of severe DNA damage and pathological stress, overexpression of PAR polymerase (PARP)-1 causes excessive production of PAR, which releases apoptosis-inducing factor (AIF) from the mitochondria. AIF then translocates to the nucleus, where it induces extensive DNA fragmentation, leading to cell death [27,28,29]. This pathological mechanism, designated as parthanatos, plays a key role in tissues with high metabolic activity, such as the heart, and is involved in numerous cardiovascular diseases such as myocardial ischemia/reperfusion (I/R) injury and heart failure [30,31]. Unlike classical apoptosis, parthanatos does not depend on the activation of specific “death proteins” such as caspases. Instead, it utilizes pleiotropic proteins that perform essential functions under physiological conditions such as PARP-1, an important signaling molecule initiating DNA repair processes [32], or AIF, which is involved in normal mitochondrial respiration [28]. In cases of extreme cellular stress, this pathway is hyperactivated and then functions as an emergency program to eliminate the damaged cells. Studies on rats exposed to normobaric or hypobaric hypoxia showed increased ROS/RNS generation in the myocardium [33,34]. Further, a previous study on rats exposed to normobaric hypoxia over three days showed a significant increase in NT, PARP-1, PAR, and AIF in the heart [1], indicating that this pathway is also implicated in the myocardial injury induced by exposure to systemic hypoxia. Strong sympathetic activation or administration of high doses of NE can also increase the formation of ROS/RNS, activate PARP-1 and induce apoptosis and necrosis [35,36,37,38], which may further promote the hypoxic myocardial damage.

The main aim of the present study was to investigate the effects of a three-day normoxic recovery on cardiac function and on markers of hypoxic myocardial injury. As ischemia-induced damage to myocardial cells is often deteriorated after reperfusion [39,40,41], we hypothesized that oxidative/nitrosative stress and the associated myocardial damage, including parthanatos, might persist after return to normoxia. This leads to the second question as to whether heart function improves or remains impaired after reoxygenation. The third question was related to the contribution of NE administration to myocardial function and myocyte injury, with particular focus on oxidative/nitrosative stress, PAR formation and AIF release. In a previous study, we investigated the effects of three days of hypoxia plus adrenergic blockade on the heart [1]. The present study aims to supplement this work by examining the effects of sympathetic overstimulation. NE infusion is an established model for the development of cardiac hypertrophy and remodeling [42,43]. Therefore, we chose this model here as well, even though external NE administration differs in some respects from the effects of true sympathetic overactivity. An important difference is that NE infusion has predominantly systemic α_1_-adrenergic vasoconstrictor effects while β-adrenergic effects are moderate to mild [44]. Here, we hypothesized that NE administration would aggravate hypoxia-induced myocardial damage and parthanatos. Finally, we investigated whether and to what extent myocardial function and myocardial injury recede after this treatment, followed by three days of normoxic recovery and withdrawal of NE.

## 2. Materials and Methods

### 2.1. Animal Model

All experiments were conducted on 98 female Sprague Dawley rats supplied by Charles River (Sulzfeld, Germany). At the beginning of the study, the body weight (BW) of the animals was 242.8 ± 1.9 g, corresponding to an age of about 10–12 weeks. All animal protocols were approved by the Federal State Agency (Landesdirektion Sachsen, protocol number TVV 46/18). The experiments were performed according to the Guide for the Care and Use of Laboratory Animals, published by the National Institutes of Health, and with the “European Convention for the Protection of Vertebrate Animals used for Experimental and other Scientific Purposes” (Council of Europe No 123, Strasbourg 1985).

### 2.2. Study Protocol

**Figure 1:**
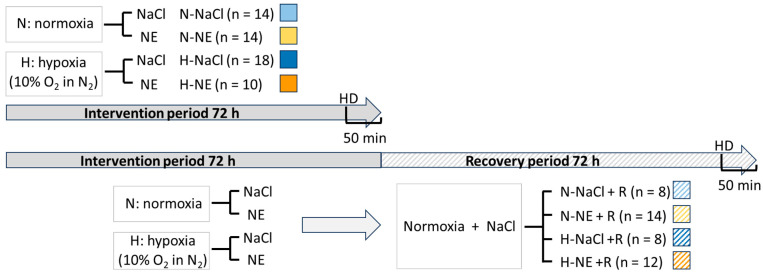
Experimental protocol: Upper part: Four groups of rats were exposed only to the 72-h intervention period, i.e., exposure to room air (normoxia, N) or normobaric hypoxia (H, 10% O_2_ in N_2_) with infusion of 0.9% saline (NaCl) or norepinephrine (NE, 0.1 mg kg^−1^ h^−1^). Lower part: Four other groups of rats were exposed to the intervention period, followed by a 72-h recovery period with exposure to room air (normoxia, N) and infusion of 0.9% saline (NaCl). HD marks the start of hemodynamic measurements (50 min before the end of the experiment). The color and pattern codes presented here are also used in the following figures for the respective animal groups.

Animals were divided at random into two cohorts for exposure to normoxia (N, n = 50) or normobaric hypoxia (H, n = 48). The animals were housed in individual cages, which were placed in a 65 × 105 × 50 cm chamber. This chamber was ventilated, either with ambient air (for normoxic animals) or with a gas mixture containing 10% oxygen in nitrogen (for animals exposed to hypoxia). A special device prevented ambient air from entering the chamber during manipulations on the animals, thus maintaining a stable oxygen concentration of 10 ± 0.5% inside the chamber. All animals were infused via an intravenous catheter (Vygon, Aachen, Germany) using automatic pumps (Infors AG, Basel, Switzerland) at a rate of 0.1 mL h^−1^ throughout the entire experimental period. Both the normoxic and hypoxic cohorts were randomly subdivided into two groups, one infused with 0.9% sodium chloride (NaCl) solution and the other with NE (0.1 mg kg^−1^ h^−1^). This first phase of the experiment, the intervention period, lasted 72 h; after that, the experiment ended for portion of the animals of those four groups. These four subgroups were referred to as N-NaCl (n = 14), H-NaCl (n = 18), N-NE (n = 14), and H-NE (n = 10). For the remaining animals, the intervention period was followed by a 72-h recovery period during which they remained in normoxia and were infused with NaCl. These animals were labeled as N-NaCl+R (n = 8), H-NaCl+R (n = 8), N-NE+R (n = 14) and H-NE+R (n = 12) (Figure 1). The transition to the recovery period did not require any direct manipulations on the animals.

At the beginning of the experiment, we inserted the infusion catheter into the left jugular vein under anesthesia with 2% isofluran. The exposure to the hypoxic environment started immediately after catheter insertion. After waking up from anesthesia, the rats were able to move freely in their cages and had access to tap water and a rat chow diet (Altromin C100, Altromin GmbH, Lage, Germany). The oxygen concentration in the chamber, as well as sufficient availability of food and drinking water, were checked regularly.

### 2.3. Hemodynamic Measurements

About 50 min before the end of the experiment, the animals were anesthetized with an intraperitoneal injection of thiopental (Trapanal^®^ 80 mg kg^−1^; Inresa Arzneimittel, Freiburg, Germany). The animals were weighed to calculate the BW difference over the experimental period. Once a sufficient depth of anesthesia had been achieved, as verified by testing the foot-withdrawal reflex, we performed a tracheotomy and placed a polyethylene cannula into the trachea. First, we catheterized the right ventricle (RV) with a Millar^®^ (Millar Instruments, Houston, TX, USA) ultraminiature catheter pressure transducer, which was inserted through the right internal jugular vein. After measuring RV hemodynamics, a pressure–volume catheter (Millar Instruments, Houston, TX, USA) was inserted into the left ventricle (LV) via the right carotid artery. Data acquisition and analysis were performed with Power Lab and Lab Chart Software from ADInstruments (version 8.1.9, ADInstruments Europe/UK, Oxford, UK) and modified LabChart Software (version 1.0) from the ADInstruments sales department (FMI Föhr Medical Instruments GmbH, Seeheim, Germany). Parallel conductance was corrected by injection of 0.1 mL of 0.9% NaCl solution, and stroke volume (SV) was calibrated by the thermodilution method. The following variables were measured in RV and LV: systolic peak pressures (LVSP, RVSP), heart rate (HR), and the maximum rate of pressure increase (LV dP/dt max, RV dP/dt max) or decrease (LV dP/dt min, RV dP/dt min) as measures of ventricular contractility and relaxation, respectively. In the LV, we further determined stroke volume (SV), ejection fraction (EF), and stroke work (SW), as well as end diastolic pressure (LV edP) and volume (LV edV). After withdrawing the LV catheter tip into the aorta, diastolic aortic pressure (DAP) was measured to calculate mean aortic pressure (MAP). In addition, we used the thermodilution method to measure the cardiac index (CI, body mass-related cardiac output) using a thermosensitive 1.5F microprobe and a Cardiomax II computer (Columbus Instruments, Columbus, OH, USA). From this, we calculated the total peripheral resistance (TPR) by dividing MAP by CI.

Animals of the H-NaCl and H-NE groups remained in hypoxia until the completion of the hemodynamic measurements.

### 2.4. Sampling of Materials

After the hemodynamic measurements were completed, the abdominal cavity was opened, and blood was drawn from the abdominal aorta, thereby sacrificing the animals. The aortic blood was used for oximetry and blood gas analysis, which were carried out with the ABL800 BASIC blood gas analyzer (Radiometer Medical ApS, Brønshøj, Denmark). We measured the arterial saturation of oxygen (SaO_2_), the partial pressures of oxygen (pO_2_) and carbon dioxide (pCO_2_), the pH, the concentration of lactate (cLac), the concentrations of potassium (cK^+^) and sodium (cNa^+^), as well as the concentration of hemoglobin (cHb) and the hematocrit (Hct).

Then the heart was excised, and the apex was trimmed off and fixated in formalin for immunohistochemical analyses. At the end of the experiment, the leftover feed was weighed to determine the total feed consumption and to calculate the daily feed intake of the animals. Drinking water consumption was measured every day for all animals that were in the experiment for 6 days, meaning those that had completed both the intervention and recovery phases. This allowed us to directly compare the water intake during the two experimental phases in the same animals.

### 2.5. Immunohistochemistry

Immunohistochemical analysis was applied to determine markers of oxidative/nitrosative stress (NT), for DNA damage and its repair (PAR), and for the PAR-induced apoptosis (AIF) in the heart. We cut 2 μm thick sections of the cardiac apex. These slices were dewaxed, rehydrated, cooked in 0.01 M citrate buffer (pH = 6) and then blocked with bovine serum albumin to saturate unspecific bindings. The samples were incubated with the primary antibodies at 4 °C overnight. For the determination of NT, we used mouse monoclonal anti-nitrotyrosine primary antibody (1 mg/mL, dilution 1:100; product number = MAB5404; Merck-Millipore, Darmstadt, Germany). For PAR, the primary antibody was mouse monoclonal antibody IgG2a (1 mg/mL, dilution 1:200; product number = ALX-804-220; Enzo, Lörrach, Germany), and for determination of AIF, we employed mouse monoclonal antibody IgG (200 μL/mL, dilution 1:50; product number = sc-13116; Santa Cruz, Heidelberg, Germany). On the second day, the samples were washed, and the horseradish peroxidase-labeled secondary antibody (goat anti-mouse antibody; dilution 1:200; product number = 12-349; Merck-Millipore, Darmstadt, Germany) was added and incubated for 1 h (AIF and PAR) or 2 h (NT) at room temperature. After another wash cycle, we applied AEC red chromogen (Enzo, Lörrach, Germany) to visualize the positive cells. Cell nuclei were counterstained with hemalum.

To conduct the microscopic examination and photography, we used the Axioimager M1 microscope from Zeiss (Carl Zeiss, Jena, Germany), together with an AxioCam MRc 5 camera and Zen Blue 3.1 software (Carl Zeiss, Jena, Germany). At least 50 photographs per animal were captured at 100× magnification for the identification of each marker. The positive areas (in µm^2^) in the pictures were measured using the ImageJ 1.54 [45] program. The samples were evaluated by two independent, blinded experimenters (A.S. and B.R.). The expression of each marker is given as the positive area related to the total area of the specimen (in percent).

### 2.6. Statistical Analysis

Statistical analyses were performed using analysis of variance (ANOVA) procedures via the software package SigmaPlot Version 16.0 (Systat Software GmbH, Erkrath, Germany) for Windows. As a first step, a Shapiro–Wilk test was conducted to ensure normal distribution. If the data were normally distributed, a one-way ANOVA with post hoc tests, according to the Holm–Sidak method was performed to compare all groups against one another. If the data were not normally distributed, we employed a Kruskal–Wallis ANOVA on ranks with a post hoc test, according to Dunn’s method. Since the experiment included various treatment factors, their main effects and interactions among the treatment factors were examined using a three-way ANOVA. We defined the factors A = environment with the levels normoxia (N) and hypoxia (H), B = infusion with the levels NaCl and NE, and C = periods with the levels intervention (INT) and intervention + recovery (INT+R). Post hoc tests were performed using the Holm–Sidak method. *p* values < 0.05 were considered significant. The results of the three-way ANOVA are presented as the *p*-values of the factor interactions. The main effects of the factors are reported only if there are no significant interactions with other factors.

### 2.7. Use of Generative Artificial Intelligence (GenAI)

We used GenAI exclusively to assist with literature research while drafting the manuscript.

## 3. Results

### 3.1. General Metabolic Situation

**Figure 2:**
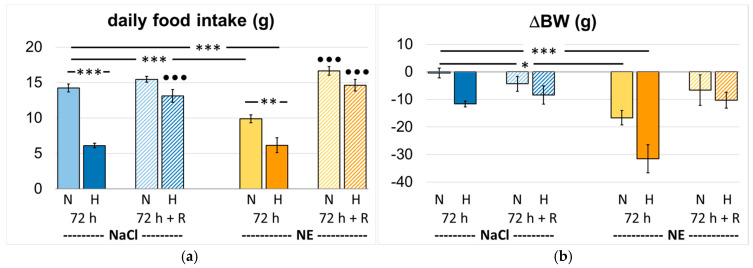
(**a**) Daily food intake in g; (**b**) change in body weight (ΔBW) in g between the first and last day of the experiment. Data are given as mean ± SEM. N, normoxia; H, hypoxia; 72 h, intervention period only; 72 h + R, intervention + recovery period; NaCl, saline infusion; NE, norepinephrine infusion. Significance marks: * *p* < 0.05, ** *p* < 0.01, and *** *p* < 0.001; significant differences vs. corresponding intervention period: ••• *p* < 0.001.

Under hypoxia, food intake was reduced by more than 50% compared to that of the normoxic control animals (*p* < 0.001, see Figure 2a). Consequently, the H-NaCl animals lost 11.7 g on average (equivalent to 5.1% of their initial body weight) during the days of hypoxia, while normoxic animals maintained their body weight (BW) as almost stable. NE infusion also reduced food intake, particularly in normoxia. The three-way ANOVA revealed significant interactions among the factors. We found significant differences in factor A (i.e., between N and H) at both levels of factor B, and significant differences in factor B (i.e., between NaCl and NE) at level N of factor A during the intervention period (*p* < 0.001; Table 1). Accordingly, BW loss during NE infusion was significantly greater, even in normoxia (16.7 g; 6.8%; *p* = 0.01), but even more so in hypoxia (32.7 g; 12.9%; *p* < 0.001). The three-way ANOVA showed significant differences between NaCl and NE in the intervention period (*p* < 0.001; Table 1). During the 72 h recovery period, food intake normalized, and the BW returned to nearly its initial level and remained only slightly below it (Figure 2).

**Figure 3:**
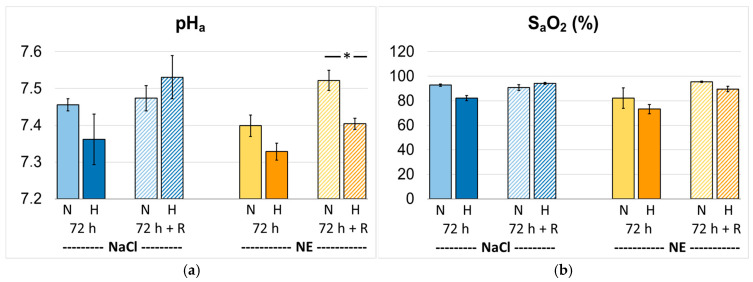
(**a**) arterial pH; (**b**) arterial oxygen saturation (SaO_2_) in %. Data are given as mean ± SEM. N normoxia; H hypoxia; 72 h intervention period only; 72 h + R intervention + recovery period; NaCl saline infusion; NE norepinephrine infusion. Significance marks: * *p* < 0.05.

Arterial oxygen saturation (SaO_2_) and pO_2_ decreased in hypoxia from 93% to ~82% and from 96 to 88 mmHg, respectively, in the NaCl-infused rats. The acid–base situation shifted in these animals towards a mildly acidotic state, with a pH of about 7.35 and a lactate concentration in the blood of 3.6 mmol/L. pCO_2_ was slightly reduced to ~33 mmHg, indicating mild hyperventilation. With NE infusion, these changes were even more pronounced. SaO_2_ was already ~82% in the normoxic NE group and decreased further to ~73% under hypoxia. For SaO_2_, we observed significant main effects in the three-way ANOVA for factors A (N vs. H: p = 0.042) and C (INT vs. INT+R: *p* < 0.001). pO_2_ also decreased slightly, but not significantly, with NE administration. pH in the N-NE group was normal (7.4), and pCO_2_ was moderately elevated (49 mmHg). Under hypoxia and NE infusion, both values decreased to 7.33 and 32 mmHg, respectively. Most of these parameters tended to normalize during the recovery period, only pCO_2_ decreased further in animals that were previously infused with NE (Figure 3 and Table 1 and Table 2).

**Figure 4:**
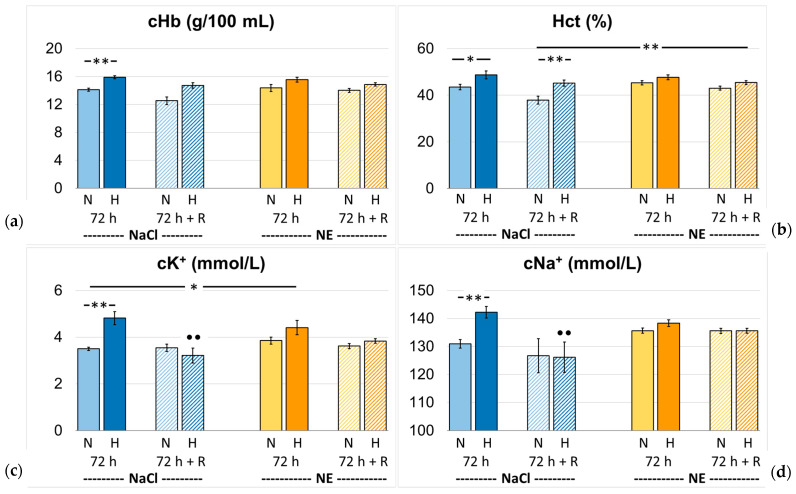
(**a**) Hemoglobin concentration (cHb) in g/100 mL; (**b**) hematocrit (Hct) in %; (**c**) K^+^ concentration (cK^+^) in mmol/L; (**d**) Na^+^ concentration (cNa^+^) in mmol/L. Data are given as mean ± SEM. N, normoxia; H, hypoxia; 72 h, intervention period only; 72 h + R, intervention + recovery period; NaCl, saline infusion; NE, norepinephrine infusion. Significance marks: * *p* < 0.05, ** *p* < 0.01; significant differences vs. corresponding intervention period: •• *p* < 0.01.

**Figure 5:**
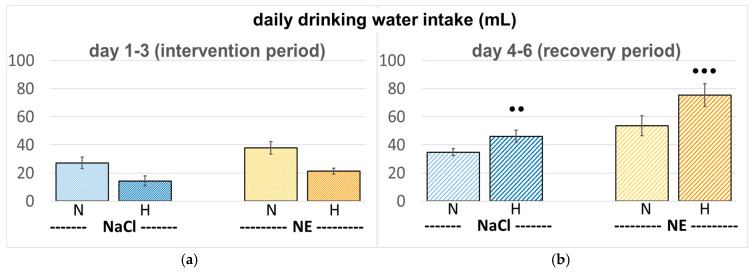
Daily drinking water intake in mL: (**a**) furing the intervention period (day 1–3); (**b**) during the recovery period (day 4–6). Drinking water consumption was measured only in the animals that were exposed to the 6-day experiment, including the intervention and recovery phases. Data are given as mean ± SEM. N, normoxia; H, hypoxia; NaCl, saline infusion; NE, norepinephrine infusion. Significant differences vs. corresponding intervention period: •• *p* < 0.01; ••• *p* < 0.001.

Three days of hypoxia induced a significant increase in hemoglobin concentration (cHb) and hematocrit (Hct), indicating that acclimatization to hypoxia is taking place. For cHb, the three-way ANOVA revealed significant main effects for factors A (N vs. H: *p* < 0.001) and C (INT vs. INT+R: *p* < 0.001). With NE infusion, both cHb and Hct were already slightly elevated in normoxia, but further increased under hypoxia to a similar level as that resulting from NaCl infusion. After three days of normoxic recovery, they showed a tendency to return to normal levels, but not to complete normalization. However, serum potassium (cK^+^) and sodium concentrations (cNa^+^) also increased during the hypoxic period and reverted after three days of recovery (Figure 4). It should be noted that the animals reduced their daily fluid intake during the period of hypoxia by about 50% (14.4 mL/d compared to 27.2 mL in normoxic animals), but compensated for this during the days of recovery. With NE infusion, the daily fluid intake was even mildly increased in normoxia, but also decreased under hypoxic conditions. During recovery, the daily fluid intake increased even further in the formerly normoxic NE animals. However, in the previously hypoxic animals, the fluid intake per day reached levels more than three times as high as those during the intervention period (76.6 mL/d compared to 21.4 mL/d; Figure 5). There was a significant difference in factor C between INT and INT+R at level H of factor A (*p* < 0.001).

The significance levels from the three-way ANOVA for the parameters described in this section are presented in Table 1.

### 3.2. Hemodynamic Results

**Figure 6:**
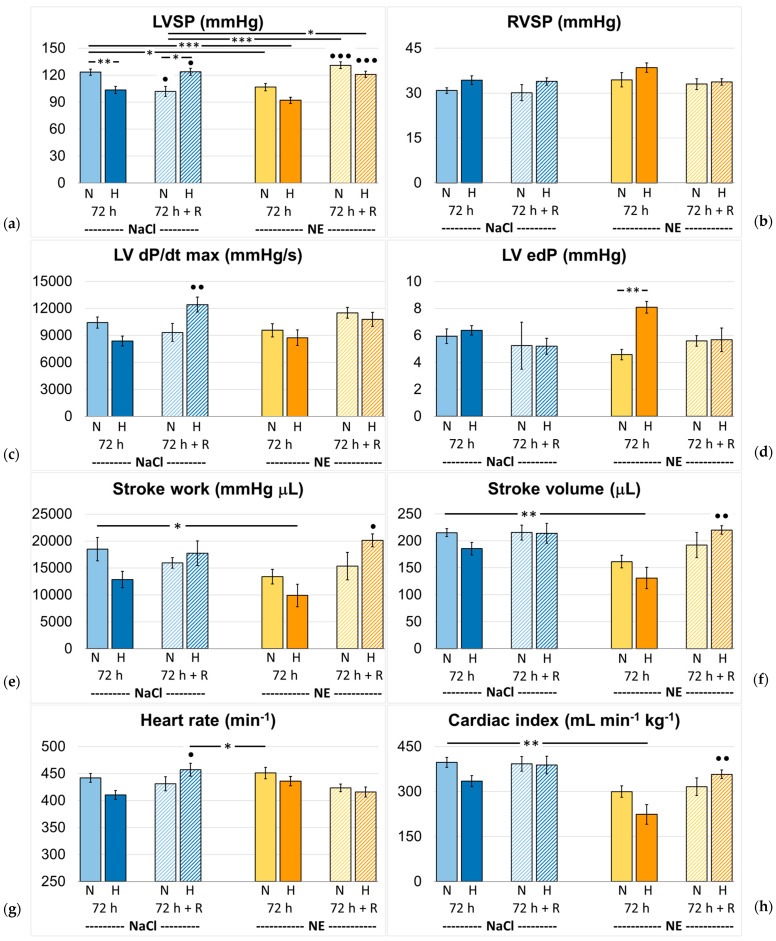
(**a**) Left ventricular systolic peak pressure (LVSP) in mmHg; (**b**) right ventricular systolic peak pressure (RVSP) in mmHg; (**c**) left ventricular maximum rate of pressure increase (LV dP/dt max) in mmHg/s; (**d**) left ventricular end-diastolic pressure (LV edP) in mmHg; (**e**) stroke work in mmHg μL; (**f**) stroke volume in μL; (**g**) heart rate in min^−1^; (**h**) cardiac index in mL min^−1^ kg^−1^. Data are given as mean ± SEM. N, normoxia; H, hypoxia; 72 h, intervention period only; 72 h + R, intervention + recovery period; NaCl, saline infusion; NE, norepinephrine infusion. Significance marks: * *p* < 0.05; ** *p* < 0.01; *** *p* < 0.001; significant differences vs. corresponding intervention period: • *p* < 0.05; •• *p* < 0.01; ••• *p* < 0.001.

Hemodynamic data are presented in Figure 6 and Table 3 and Table 4. Exposure to hypoxia deteriorated LV systolic function, as indicated by significant differences in factor A (environment, i.e., between N and H) at level INT of factor C for numerous parameters such as LVSP, SV, SW, CI, EF, and HR. A significant reduction in LV dP/dt min and an increase in LV edP at level INT demonstrate that LV diastolic function was compromised as well (Table 3). In contrast, RV function was slightly improved under hypoxia. There was a significant main effect of factor A (N vs. H: *p* = 0.019), without significant interactions with the other factors. RV dP/dtmax was slightly, but not significantly, improved by about 7%. The TPR was slightly but not significantly reduced.

NE infusion further aggravated the LV depression, even in normoxia, as particularly evidenced in LVSP showing significant interactions among all three factors, with a significant difference in factor B (NaCl vs. NE: *p* = 0.003) at level N of factor A and level INT of factor C (Table 3). Under hypoxic conditions, the deterioration in LV function was even more prominent. LVSP decreased significantly by about 25% and consequently, SV, SW and CI also decreased significantly by about 40–50% compared to the results for the N-NaCl group. For SV, there were significant differences in factor B (NaCl vs. NE) at level INT of factor C (*p* < 0.001). TPR was mildly elevated in the NE-treated animals, as indicated by a significant difference in factor B (infusion, i.e., between NaCl and NE) at level INT of factor C (*p* = 0.024). LV edP decreased in N-NE rats but increased in the H-NE group by more than one third above the level in N-NaCl animals. This is also underscored by significant differences in factor B (NaCl vs. NE) at level H of factor A (*p* = 0.041) and in factor A (N vs. H) at level NE of factor B (*p* = 0.002; Table 3). In contrast, RV function improved slightly further during NE infusion. In RVSP, we found a significant main effect of factor B (infusion; *p* = 0.04); with RV dP/dtmax and RV dP/dtmin, there were no significant main effects or interactions among the factors.

After a three-day period of normoxic recovery with NaCl infusion, most of the hemodynamic parameters were restored to the levels of the N-NaCl group or even exceeded them. This is evident from the results of the three-way ANOVA: Many parameters show significant differences in factors A and B at level INT of factor C, but not at level INT+R (see Table 3). Only HR and consequently, CI, remained slightly below the values of the N-NaCl group in the previously NE-infused animals. For HR, there was a significant interaction between factors B and C at level INT+R (*p* = 0.019).

### 3.3. Immunohistochemical Results

**Figure 7:**
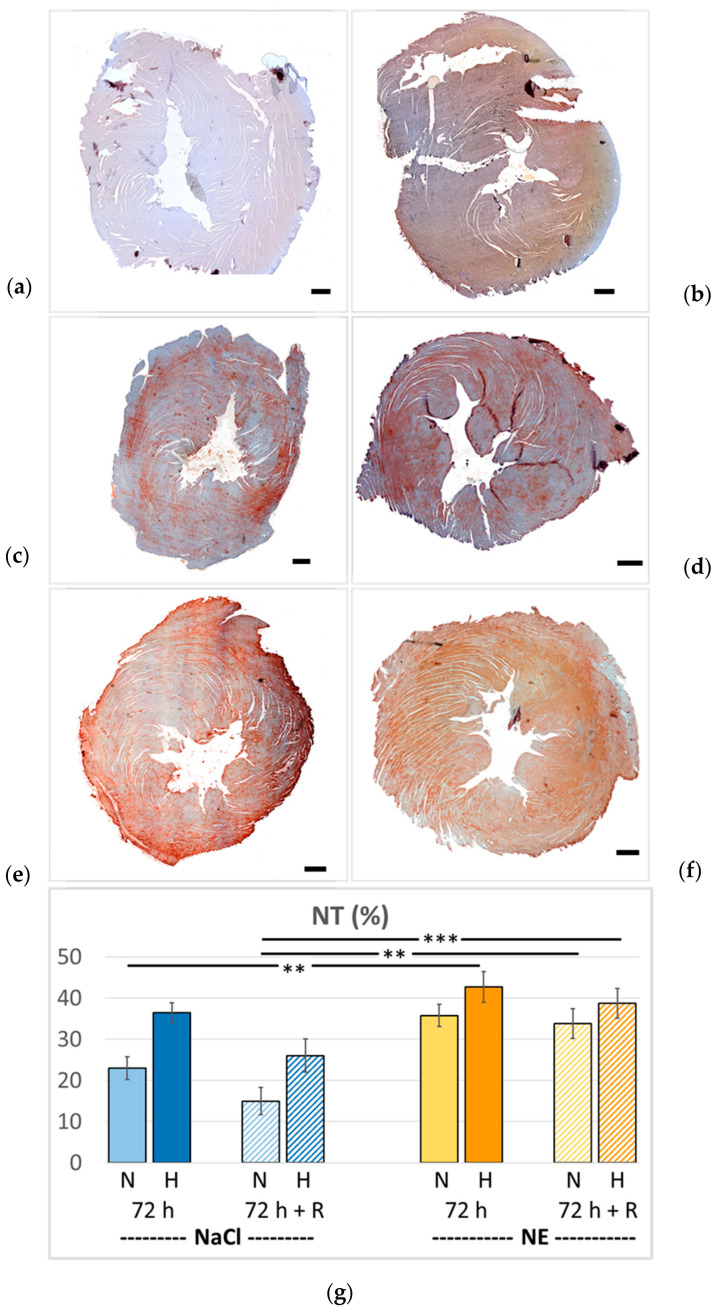
Nitrotyrosine (NT) in the heart: representative immunohistochemical images from N-NaCl (**a**), H-NaCl (**b**), N-NE (**c**), H-NE (**d**), H-NaCl+R (**e**), and N-NE+R (**f**) hearts. (**g**) Abundance of NT in the heart expressed as the percentage of positive area related to the total area of the cardiac walls. Data are presented as means ± SEM. N, normoxia; H, hypoxia; NaCl, saline infusion; NE, norepinephrine infusion. Significance marks: ** *p* < 0.01; *** *p* < 0.001. All images are shown in the same magnification (100×), with the scale bars indicating 500 µm each.

Hypoxia induced oxidative/nitrosative stress in the heart, as indicated by a significant increase in NT. The three-way ANOVA revealed a significant main effect for factor A (N vs. H: *p* < 0.001). NE infusion, however, increased NT to almost the same level, even in normoxia, and increased it further under hypoxic conditions. There was also a significant main effect for factor B (NaCl vs. NE: *p* < 0.001). Three days of recovery largely restored the NT levels in the animals that had only received NaCl infusion. Of note, in rats kept in normoxia with NaCl infusion over six days, the NT values were even lower than those after three days of this treatment. The three-way ANOVA also showed a significant main effect for factor C (INT vs. INT+R: *p* = 0.013), even though there was hardly any decrease in the NT levels after the recovery phase in those animals that were previously infused with NE (Figure 7). There were no statistically significant interactions among the factors.

**Figure 8:**
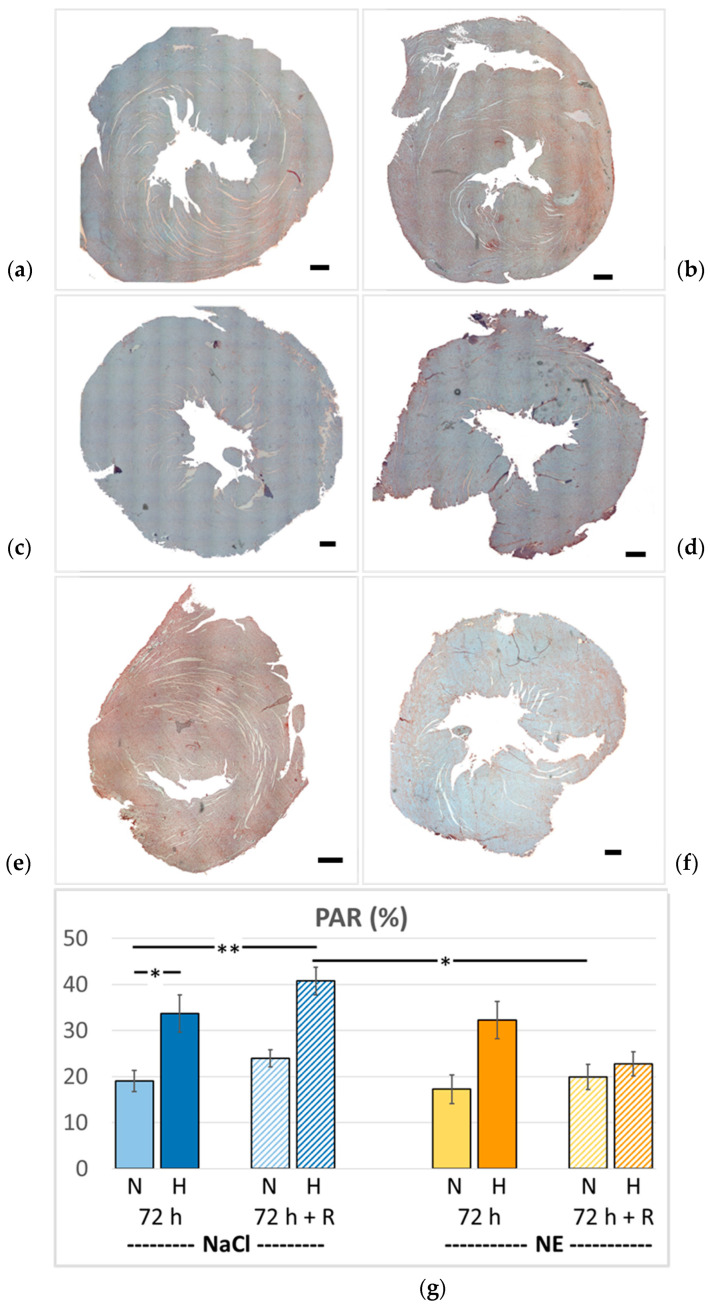
Poly(ADP-ribose) (PAR) in the heart: representative immunohistochemical images from N-NaCl (**a**), H-NaCl (**b**), N-NE (**c**), H-NE (**d**), H-NaCl+R (**e**), and N-NE+R (**f**) hearts. (**g**) Abundance of PAR in the heart expressed as the percentage of positive area related to the total area of the cardiac walls. Data are presented as means ± SEM. N, normoxia; H, hypoxia; NaCl, saline infusion; NE, norepinephrine infusion. Significance marks: * *p* < 0.05; ** *p* < 0.01. All images are shown in the same magnification (100×), with the scale bars indicating 500 µm each.

Moreover, we found a significant increase in the levels of PAR under hypoxia. The extent of hypoxic PARylation was in a similar range with levels for NaCl and NE infusion. In the three-way ANOVA, we found a significant main effect of factor A (N vs. H: *p* < 0.001) but no interactions of factor A with factor B or C. After the recovery period, the PAR levels receded in the animals previously exposed to NE plus hypoxia, but they were even higher in those animals that had received NaCl during the intervention phase (Figure 8). There was a significant interaction between factors B and C: The difference in factor C (INT vs. INT+R) was significant at level NaCl of factor B (*p* = 0.047), and the difference in factor B (NaCl vs. NE) was significant at level INT+R of factor C (*p* < 0.001).

**Figure 9:**
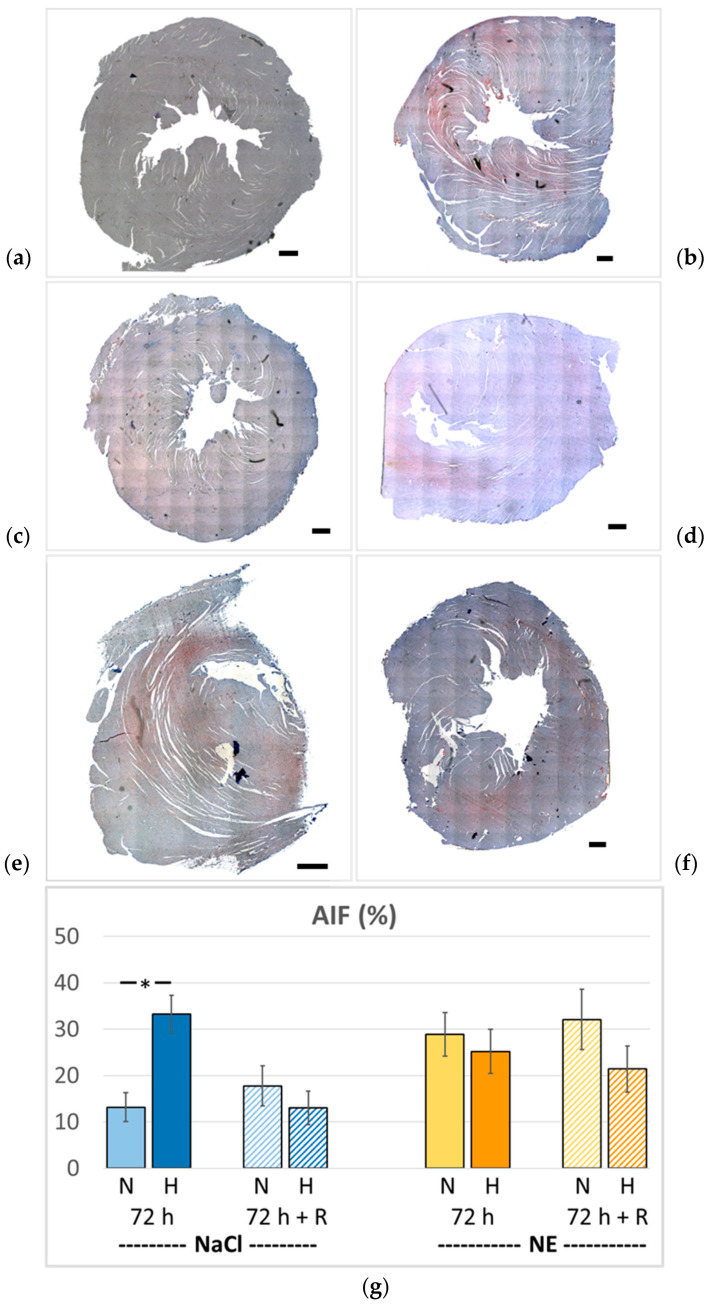
Apoptosis-inducing factor (AIF) in the heart: representative immunohistochemical images from N-NaCl (**a**), H-NaCl (**b**), N-NE (**c**), H-NE (**d**), H-NaCl+R (**e**), and N-NE+R (**f**) hearts. (**g**) Abundance of AIF in the heart expressed as the percentage of positive area related to the total area of the cardiac walls. Data are presented as means ± SEM. N, normoxia; H, hypoxia; NaCl, saline infusion; NE, norepinephrine infusion. Significance marks: * *p* < 0.05. All images are shown in the same magnification (100×), with the scale bars indicating 500 µm each.

Finally, we observed a significant increase in AIF under hypoxia in NaCl-infused rats (*p* = 0.017), which completely regressed after three days of normoxic recovery. With NE infusion, however, AIF was significantly elevated, even in normoxia, and did not further increase under hypoxic conditions. We found a significant difference in factor B (NaCl vs. NE: *p* = 0.002) at level N of factor A. In the recovery period, the AIF levels declined partially in the formerly hypoxic animals, but increased even more in animals that were previously exposed to NE and normoxia (Figure 9). There was a significant difference in factor C (INT vs. INT+R, *p* = 0.012) at level H of factor A. Of note, the immunohistochemical images showed a rather diffuse distribution of AIF within the cell, with no clear localization in the nucleus.

## 4. Discussion

### 4.1. General Metabolic Situation

Exposure to a hypoxic environment with atmospheric O_2_ levels or pO_2_ values that are only half those found at sea level leads to arterial hypoxemia, resulting in insufficient oxygen supply to the tissues. This, in turn, means that the body must increasingly rely on anaerobic glycolysis to meet its energy needs [18,46]. The accumulation of lactate leads to metabolic acidosis. Both hypoxemia and metabolic acidosis cause an increase in ventilation, which counteracts the acidosis. In awake rats, the mean arterial pH (pH_a_) is slightly higher, at 7.47 ± 0.02, while the mean arterial pCO_2_ is slightly lower, at 34.5 ± 3.0 mmHg, than that in humans [47]; the values observed in the N-NaCl group thus correspond well to these normal values. In contrast, pH values of 7.35 or lower, as measured in the hypoxic animal groups (H-NaCl; H-NE), together with the elevated lactate levels, indicate metabolic acidosis. The pCO_2_ levels of approximately 33 mmHg measured in these groups are slightly diminished, indicating mild hyperventilation, which is caused by hypoxia on the one hand, but which also represents a respiratory compensation for metabolic acidosis. After 3 days of normoxic recovery, pH values have shifted toward alkalosis, which may result from hyperventilation that persists after return to normoxia as part of a deacclimatization reaction [48]. This post-hypoxic hyperventilation largely subsides within 24 h [49], which could explain the normal pCO_2_ levels in the H-NaCl group. As hypoxia-induced sympathetic activation also persists over several days after the return to normoxia [14], we would expect that hyperventilation during the recovery phase following NE infusion would be prolonged and more pronounced than after NaCl infusion, resulting in a significant reduction in pCO_2_ levels in the NE+R groups.

Despite the reduced energy supply, appetite and food uptake are reduced under hypoxic conditions [50], and this has also been observed in travelers to high altitudes [51,52]. We assume that reduced feed and water intake are major causes for the BW loss of hypoxia-exposed animals. However, there must be other factors contributing to the BW loss, as also demonstrated by a comprehensive meta-analysis [53]. While food intake during NE infusion was only little (in normoxia) or not at all reduced (in hypoxia) compared to that in the respective NaCl groups, BW loss was significantly greater than that with NaCl infusion. We suspect that increased physical activity and stress in NE-infused rats are the reasons for the greater BW loss of these animals, a conclusion supported by the greater BW loss in humans during exposure to moderate or high altitudes when combined with physical activity compared to the results during physical inactivity [53]. Another factor contributing to the reduction in BW is a reduction in plasma volume [54,55]. A reduced plasma volume means a reduced load for the heart, which improves oxygen supply to the tissues while at the same time reducing the strain on the heart. The main mechanism of this hypoxia-induced fluid loss is increased urine excretion, also referred to as acute hypoxic diuretic response [56,57], but it is enhanced by reduced fluid intake [54], as we also observed in the animals in the present study during hypoxia exposure. Plasma volume reduction, which can already exceed 10% within the first 24 h, is an early response during adaptation to high altitude or systemic hypoxia [55], contributing to an early increase in cHb and Hct. A sustained increase in O_2_ transport capacity is triggered by the increase in erythropoiesis mediated by HIFs and erythropoietin and begins after a few days of hypoxia [58,59]. The elevated values of cHb, Hct, cK^+^ and cNa^+^, combined with reduced water intake in the hypoxic animal groups, demonstrate that these adaptive responses also occurred in the hypoxic animals in the present study. An earlier study in rats exposed to hypoxia for 6 days demonstrated that cHb continues to rise significantly with prolonged exposure to hypoxia [3].

### 4.2. Hemodynamic Situation

Previous studies revealed that hypoxia significantly impairs LV systolic function, as reflected in reduced values of LVSP, LV dP/dt max, MAP, DAP, SV, SW, and CI. It is well known that the anesthetic thiopental can reduce the contractile force of the heart and cause a drop in blood pressure, accompanied by reflex tachycardia. Treating hypoxic and/or NE-infused animals in the same manner as normoxic control animals and comparing them with the controls allows the treatment effects to be distinguished from any potential cardiodepressive effects of thiopental. LV depression starts after just 6 h of hypoxia exposure and persists with continued hypoxia [1,2], as confirmed by the results of the present study. This functional impairment is largely based on an energetic depletion of myocardial cells resulting from the shift in their energy metabolism to anaerobic glycolysis, which is less ATP-efficient [16,18,60]. LV diastolic function is generally even more sensitive than systolic function to a lack of energy, as the return of Ca^2+^ from the cytoplasm to the sarcoplasmic reticulum is delayed, and this impairs LV relaxation [61]. The resulting increase in stiffness is reflected in the increased LV edP in the hypoxic animals in this study. In addition, oxidative and nitrosative stress and apoptosis cause myocardial damage [1], which may further impair myocardial function, as discussed in more detail below. The reduction in SV is exacerbated by hypovolemia, which leads to diminished venous return and reduced end-diastolic filling [62]. A decrease in HR, which is promoted by elevated cK^+^ in the blood, also contributes to the deterioration of LV function [63]. An earlier study in rats exposed to hypoxia for up to 24 h showed that the hypoxia-induced sympathetic activation was relatively weak and was not sufficient to prevent the deterioration of LV function [2]. This is further supported by the fact that adrenergic blockade only caused a slight further deterioration in LV function under hypoxic conditions [1]. In contrast, RVSP was not impaired under hypoxic conditions but actually increased slightly, even though hypoxia-induced myocardial damage due to oxidative/nitrosative stress, PARylation, and apoptosis was similarly pronounced in both the RV and LV [1]. One possible cause of the slightly elevated RVSP could be hypoxic pulmonary vasoconstriction, since the resulting increase in pulmonary vascular resistance requires the RV to generate higher pressure. In the systemic circulation, however, TPR did not increase because hypoxia has a vasodilatory effect on systemic arteries [64]. Due to the lower pressures in the pulmonary circulation, the systolic work of the RV is only about 1/5 to 1/7 of the stroke work of the LV, so that hypoxic myocardial damage has a less pronounced effect on the pumping function of the RV than on that of the LV. Another explanation for the different responses of the two ventricles to hypoxia can be found in the differing densities of adrenergic receptors between LV and RV and their response to hypoxia [65]. In addition, transcriptomic studies have shown that under hypoxic conditions, numerous genes are expressed at higher levels in the RV than in the LV, suggesting that the RV may be better adapted to hypoxic conditions than is the LV [66]. 

However, a weak hypoxic sympathetic activation cannot be the sole explanation to account for the hypoxic LV depression. Even with additional NE infusion, systolic LV function did not improve. Although the decline in HR was prevented, SW, SV and CI actually decreased even further under hypoxia plus NE infusion. In contrast, sympathetic vasoconstrictor effects were clearly pronounced, as reflected in the increase in TPR. This may also have contributed to the depression of LV systolic function under NE infusion. These findings are in line with observations in humans at high altitude [10]. Diastolic function also further deteriorated with NE administration and hypoxia, as evidenced by the significantly increased LV edP. We assume that a lack of energy is the primary cause of the deterioration in LV function in this context, since the administration of NE disproportionately increases myocardial oxygen consumption—a phenomenon also known as the “oxygen-wasting effect of norepinephrine” [67]. This effect is already evident in normoxia, as evidenced by reduced values of SW, SV, and CI, and becomes even more pronounced under hypoxia. The increased NE clearance under hypoxia might also have contributed to the reduced LV function, despite the NE administration [9]. Additionally, the NE effects decrease with longer duration of infusion due to the downregulation of β-adrenoceptors [68]. Finally, heart cell damage caused by NE also contributes to functional decline. In human pathology, Takotsubo syndrome is a well-known condition in which excessive release of catecholamines leads to nitrosative stress, causing a life-threatening reduction in myocardial contractility that can result in acute heart failure [69,70]. In a rat model, it was demonstrated that injection of the β-adrenergic agonist isoproterenol led to an intramyocardial accumulation of NT at levels approximately four times higher than those in control animals [71]. In contrast, the function of the RV is not impaired by NE administration; in fact, it is slightly improved. This observation has been repeatedly made in earlier rat studies [42,43,44]. One possible explanation for this could be that the distribution of α- and β-adrenergic receptors differs between the two ventricles and is regulated differently in the LV and RV during NE infusion. The NE-mediated regulation of adrenergic receptors in the two ventricles is not identical to the changes in the receptors induced by hypoxia [65].

The assumption that energy depletion is the primary cause of LV depression under hypoxic conditions is strongly supported by the finding that the functional impairment resolved completely upon return to normoxia, that is, once oxygen supply was restored. In the animals that had received NE during the intervention phase, most LV functional parameters also recovered completely. LVSP and LV dP/dt max even rose to levels higher than those of the normoxic control animals. Only HR and CI remained slightly reduced after three days of recovery. This can be explained by several mechanisms. First, a re-expansion of plasma volume following the end of hypoxic exposure can lead to increased ventricular filling. This mechanical stimulus, combined with myocardial damage caused by the preceding hypoxia and NE infusion, can trigger the Bezold–Jarisch reflex, in which stimulation of ventricular mechanoreceptors and chemoreceptors causes an increase in parasympathetic activity and a decrease in sympathetic activity [72,73,74]. Such a shift in the sympatho–vagal balance can also be triggered by desensitization of the arterial baroreflex induced by sustained adrenergic stimulation, e.g., via NE infusion [75,76] and the subsequent rise in LVSP and MAP during the recovery phase [77,78]. Finally, the NE infusion leads to the downregulation of β-receptors [68,79], a process that can be further exacerbated by hypoxia [15], resulting in reduced cardiac sensitivity to sympathetic activity after the NE infusion is discontinued.

### 4.3. Immunohistochemical Signs of Myocardial Damage

In addition to the direct effect of reduced ATP production, cardiac function is also impaired by hypoxia-induced damage to myocardial cells caused by oxidative/nitrosative stress. After three days of hypoxia, the proportion of NT-positive myocardial tissue increased significantly by >50% when compared to that of normoxic animals. This increase in NT was accompanied by similar or even greater increases in PAR and AIF in the myocardium. These data confirm previous findings that hypoxia leads to increased peroxynitrite production, which in turn results in enhanced PARylation and AIF-dependent apoptosis [1]. Furthermore, activation of PARP-1 causes depletion of nicotinamide adenine dinucleotide (NAD^+^) and ATP, thus further exacerbating myocardial cell stress [27,32].

It is well known that cardiomyocyte injury not only persists after hypoxia but may paradoxically worsen during reoxygenation due to I/R injury. I/R injury is a typical complication occurring in the treatment of various cardiovascular diseases such as myocardial infarction when myocardial perfusion is restored after a period of ischemia, leading to further mitochondrial injury and aggravation of myocardial damage [80,81,82]. While ischemia typically causes more severe, focal cellular damage, hypoxia—in which blood flow is generally maintained—tends to impose a diffuse metabolic burden on tissues and organs. Reoxygenation following prolonged systemic hypoxia can induce oxidative stress, calcium dysregulation, and mitochondrial dysfunction in cardiomyocytes, resembling mechanisms of I/R injury. A study on isolated rat heart mitochondria demonstrated that reoxygenation after exposure to hypoxia, but not hypoxia alone, increased mitochondrial calcium concentration and peroxynitrite production [41]. Following ischemia or hypoxia, the production of ROS, including peroxynitrite, increases significantly immediately after reoxygenation or reperfusion and then decreases slightly [83,84,85]. However, peroxynitrite and/or NT levels remain elevated and can be detected in the tissue for hours up to several days [86,87]. This increase in ROS/RNS and the resulting damage to mitochondria and DNA lead to the activation of PARP in the reperfused myocardium. Numerous studies in various experimental models involving hypoxia/reoxygenation or coronary artery occlusion/reocclusion have shown that pharmacological inhibition or genetic deletion of PARP significantly improved the outcome of myocardial I/R injury (reviewed in [32]). In the present study as well, PARP-1 activation persisted after three days of normoxic recovery, at least in those animals that had consistently been infused with NaCl, as evidenced by the PAR levels, which were even higher than those at the end of the intervention period. Typically, I/R injury is associated with a decrease in LV contractile function [82], which was not observed in our results—on the contrary, after three days in normoxia, LV function recovered completely. We suspect that the myocardial damage caused by hypoxia/reoxygenation in our experiment was not overly severe, and that, as a result, LV function recovered relatively quickly, whereas myocardial damage takes a longer time to resolve. The persistently high levels of NT and PAR after three days of recovery may possibly be due to the arbitrary end of the observation period. We cannot draw a clear conclusion regarding the further development or regression of myocardial damage based on the available results. The statistically significant difference between NaCl- and NE-treated animals during the recovery phase suggests that pretreatment of the animals influences the temporal dynamics of PARP-1 activity; however, this would need to be confirmed by experiments involving multiple measurement time points (see Section 4.4). A key function of PARP-1 is its involvement in the translocation of AIF from the mitochondria to the nucleus, which leads to DNA fragmentation and ultimately, to cell death [27,28,29]. However, the animals of the H-NaCl+R group showed a complete return to baseline AIF levels in the heart after three days of normoxia. This may suggest that the damage to myocardial cells caused by hypoxia and subsequent reoxygenation was most likely reversible and would support the idea that oxidative/nitrosative stress—and ultimately PARP-1 activation as well—could subside following a prolonged period of normoxic recovery. Under moderate genotoxic stress, the activation of PARP-1 facilitates the repair of DNA damage without directly leading to cell death. Thus, sustained PARP activation could enable the survival of the myocardial cell [32,88]. Another possible explanation for the regression of AIF and for its rather diffuse distribution within the cell could involve the activation of anti-apoptotic signals, such as HSP70 or AKT2, which inhibit the translocation of AIF into the cell nucleus [89,90,91]. However, the decrease in AIF despite persistently elevated PAR levels can also be explained by the different temporal dynamics of the two proteins. An elevated PAR level reflects ongoing PARP-1 activity in response to persistent DNA damage, which can remain elevated over time, thus indicating sustained cellular stress. In contrast, AIF release is a transient event that occurs during the early phase of mitochondrial injury [88,92]. At later stages of apoptosis, detectability of AIF can decrease due to the clearance of apoptotic cells or the relocation of AIF to the mitochondria [93,94]. Overall, the results suggest that the reduced oxygen supply and the associated energy depletion and acidosis were the primary mechanisms underlying hypoxia-induced LV depression. The complete recovery of LV function after reoxygenation suggests that oxidative stress, PAR formation, and apoptosis, although involved in myocardial injury, did not cause sustained myocardial damage.

NE administration led to a significant increase in NT levels, even under normoxic conditions, and this effect was further amplified under hypoxic conditions. NT levels did not return to baseline, even after three days of recovery under normoxic conditions and NaCl infusion. AIF showed a similar trend: AIF levels were also significantly higher with NE in normoxia than in normoxic control animals and remained at this level after discontinuation of the NE infusion. Only after prior combined exposure to hypoxia and NE infusion did AIF levels decrease moderately. It is well known that hypoxic sympathetic activation at high altitude persists for several days after returning to normoxia [14]. Prolonged, intense sympathetic activation leads to oxidative stress and apoptosis in the myocardium through persistent stimulation of β_1_-adrenoceptors, but also via α-adrenergic receptors. In this process, NE activates intracellular signaling cascades that remain active even after NE is withdrawn. These reactive intermediates sustain ROS generation and apoptosis [36,37,95,96]. This pathway mediated by adrenergic stimulation is relevant in numerous cardiac conditions, such as Takotsubo syndrome, heart failure, and I/R injury [70,97,98,99,100]. Interestingly, PAR levels did not rise higher during NE infusion—neither under normoxic nor hypoxic conditions—than those in the corresponding NaCl-infused groups. In the H-NE+R group, PAR levels even returned to near-control levels. These results clearly demonstrate that NE is not a significant enhancer of PAR-induced apoptosis. In addition to PAR, other factors can also promote the release of AIF from the mitochondria, e.g., activation of Bcl-2 proteins, such as Bax or Bak, or of mitochondrial calpain, as well as of caspases [93,101,102]. Cell culture studies on cardiomyocytes and cardiac fibroblasts showed that NE induces apoptosis via various pathways, such as the overexpression of proapoptotic Bcl-2 proteins, c-Jun N-terminal kinase (JNK), or tumor necrosis factor (TNF)-α; however, these pathways primarily involve the activation of caspases [35,103,104]. Cardiac pathologies are usually associated with chronic severe sympathetic activation and primarily cause classic caspase-dependent apoptosis [100,105,106]. In cardiomyocytes, NE-induced ROS generation leads to mitochondrial damage and activation of mitochondrial apoptotic signals [36,70,95,106], resulting in the release of AIF from the mitochondria, even without PARP-1 overactivation. NE thus induces ROS/RNS-dependent cell damage, which, however, cannot be classified as parthanatos. Generally, the results from the NE-treated groups show that NE intensifies and prolongs mitochondrial stress, which could explain the impaired LV function observed with NE administration. Particularly under hypoxia, the mismatch between the reduced O_2_ supply and the increased energy demand caused by the NE-induced stress, as also reflected in a rise in HR, is exacerbated, leading to a significant reduction in LVSP, SV, and CI. On the other hand, the complete recovery of LV function after three days of recovery in normoxia and without further NE administration suggests that NE-induced cell damage and apoptosis were rather moderate and reversible and that hypoxic damage was scarcely exacerbated by NE administration.

### 4.4. Limitations of the Study

Although the components of PARP-1-dependent cell death (parthanatos)—such as oxidative/nitrosative stress, PAR, and AIF—increased significantly under hypoxia and/or NE administration, the results showed that the deterioration in LV function was reversible, suggesting that parthanatos played a relatively minor role in the present experimental setup. However, one of the main limitations of this study is that we have no measure of the extent of cell death or of the extent to which other mechanisms of cell damage or cell death such as classical (caspase-dependent) apoptosis might have contributed to the observed functional impairments. Future studies should include measurements of caspase activation and cell viability to obtain a clearer picture of hypoxia- and NE-mediated cardiac damage. Furthermore, it must be considered that the development of damage caused by hypoxia and reoxygenation is a gradual process. Determining relevant markers at a single, arbitrarily chosen point in time cannot adequately reflect this process. Repeated measurements at different stages of the exposure phase and above all, during the recovery phase, provide a more accurate picture of the actual extent of myocardial damage and should therefore be employed in future studies.

The reversibility of the functional impairment observed in this experiment suggests that reversible myocardial damage, resulting from myocardial energy depletion, was the predominant mechanism. An assessment of energy status, including the simultaneous determination of ATP, ADP, and adenosine monophosphate (AMP), as well as the measurement of phosphocreatine (PCr), would confirm this hypothesis and should be incorporated into future studies.

Another limitation of this study is that the present experiments were performed on female rats only. The intention behind this choice was to ensure comparability with the results of earlier studies of our group [1,2,3,44]. A comparative study on rats demonstrated that blood pressure and heart rate were similar in adult male and female rats under both normoxia and normobaric hypoxia conditions [107]. However, the hearts of male and female organisms in humans and in many animal species, including rats, exhibit numerous structural, functional, and cellular differences [108,109]. Studies in mice indicated that female animals have a better tolerance for hypoxia than do males. It was shown that chronic hypoxia induced a reduction in HR and in daytime activity in male but not in female mice. In addition, male mice exhibited stronger RV hypertrophy than did females [110]. Another study demonstrated that the hearts of male and female animals employ different mechanisms to cope with oxidative stress, with female hearts proving to be more resistant to I/R injuries than are male hearts [111]. These findings are in line with other animal studies and clinical observations in patients [98,112]. Given the differences in hypoxia tolerance between male and female animals, caution is warranted when drawing generalizations. It is possible that the present experiment might have caused more severe LV dysfunction and myocardial damage in male rats than that which we observed in female animals.

## 5. Conclusions

Our results show that the deterioration in LV function induced by three days of hypoxia exposure was completely reversed after three days of normoxic recovery, even though signs of oxidative/nitrosative stress and ongoing PARP-1 activity persisted. This suggests that—unlike in cases of I/R injury—myocardial damage caused by hypoxia and reoxygenation was relatively mild and above all, reversible. Based on this, we speculate that cell death due to parthanatos plays a minor role. This conjecture is mainly supported by the significant regression of AIF during the recovery phase following hypoxia, even though the available results cannot provide conclusive proof of this assumption. The observed functional decline is partly due to general adaptations to the challenge of hypoxia exposure; however, it is primarily regarded as a consequence of myocardial energy depletion and can be completely reversed by restoration of the O_2_ supply. NE administration exacerbated mitochondrial stress, which also persisted after the cessation of exposure to NE and hypoxia. Nevertheless, the functional impairment resolved completely, even under these conditions, indicating that additional NE administration (as a model of strong sympathetic activation) mainly causes reversible cellular damage and does not exacerbate parthanatos. The results of this study indicate that myocardial injury due to generalized hypoxia and subsequent reoxygenation differs from I/R-induced damage. These findings are not only relevant for individuals in a hypoxic environment but may also have prognostic significance for patients with hypoxemia, although it must be taken into account that the hearts of men and women exhibit a wide range of differences in their responses to hypoxia [112,113].

## Figures and Tables

**Table 1:** Three-way ANOVA results for the general condition parameters.

Three-Factor Interactions	A × B interactions (Within Factor C)								A × C and B × C Interactions			
Differences in factors	A (within B) N vs. H				B (within A) NaCl vs. NE				A (within C) N vs. H		B (within C) NaCl vs. NE	
Level of the other factors	B: NaCl		B: NE		A: N		A: H		C: INT	C: INT+R	A: N	A: H
A × B interactions at level INT of factor C (no sign. A × B interactions at level INT+R)												
Food/d	<0.001		<0.001		<0.001		0.957		-----	0.004	-----	0.066
pH	0.116								0.022	0.385	0.201	0.262
A × B interactions at level INT+R of factor C (no sign. A × B interactions at level INT)												
K^+^	0.279		0.393		0.783		0.036		<0.001	-----	0.900	-----
**Two-factor interactions only**												
Differences in factors	A (within B) N vs. H		B (within A) NaCl vs. NE		A (within C) N vs. H		C (within A) INT vs. INT+R		B (within C) NaCl vs. NE		C (within B) INT vs. INT+R	
Level of the other factor	B: NaCl	B: NE	A: N	A: H	C: INT	C: INT+R	A: N	A: H	C: INT	C: INT+R	B: NaCl	B: NE
ΔBW	0.754				0.088				<0.001	0.57	0.94	<0.001
pCO_2_	0.628				0.004	0.945	0.001	0.857	0.128	0.013	0.69	<0.001
Hct	<0.001	0.054	0.006	0.722	0.558				0.192			
Na^+^	0.286				0.056				0.884	0.001	<0.001	0.577
Water intake	0.674				0.013	0.006	0.055	<0.001	0.064			

The table shows the *p* values for the interactions between the factors. ----- Significance not calculated because it is included in the three-factor interaction. For SaO_2_, pO_2_, cLac, and cHb, there were no significant interactions.

**Table 2:** Oximetry and blood gas assessment.

		NaCl				NE		
	N-NaCl	H-NaCl	N-NaCl+R	H-NaCl+R	N-NE	H-NE	N-NE+R	H-NE+R
pO_2_ (mmHg)	95.6 ± 2.4	88.0 ± 2.7	94.3 ± 6.9	99.8 ± 4.5	80.5 ± 9.6	76.5	105.8 ± 3.8	86.9 ± 6.6
pCO_2_ (mmHg)	37.4 ± 1.8	32.6 ± 2.9	38.1 ± 2.6	35.0 ± 1.8	48.9 # ± 5.1	32.2 ± 2.1	24.7 ••• ± 3.5	28.3 ± 7.1
cLac (mmol/L)	0.95 ± 0.35	3.60 ± 1.09	2.97 ± 1.11	3.01 ± 0.85	2.77 ± 0.37	3.79 ± 1.37	2.41 ± 0.26	2.74 ± 0.56

Partial pressures of oxygen (pO_2_) and carbon dioxide (pCO_2_); concentration of lactate (cLac). Data are presented as mean ± SEM. Significance marks: significant difference vs. H-NE: # *p* < 0.05; significant difference vs. corresponding intervention period: ••• *p* < 0.001.

**Table 3:** Three-way ANOVA results for the HD parameters.

Three-Factor Interactions	A × B interactions (Within Factor C)								A × C and B × C Interactions			
Differences in factors	A (within B) N vs. H				B (within A) NaCl vs. NE				A (within C) N vs. H		B (within C) NaCl vs. NE	
Level of the other factors	B: NaCl		B: NE		A: N		A: H		C: INT	C: INT+R	A: N	A: H
A × B interactions at level INT of factor C (no sign. A × B interactions at level INT+R)												
LVSP	<0.001		0.012		0.003		0.036		-----	0.003	-----	0.003
LV dP/dt max	-----		-----		-----		-----		0.04	0.14	0.723	0.734
DAP	<0.001		<0.001		0.002		0.002		-----	0.187	-----	0.035
MAP	<0.001		0.003		0.004		0.01		-----	0.176	-----	0.01
TPR	-----		-----		-----		-----		0.366	-----	0.024	-----
**Two-factor interactions only**												
Differences in factors	A (within B) N vs. H		B (within A) NaCl vs. NE		A (within C) N vs. H		C (within A) INT vs. INT+R		B (within C) NaCl vs. NE		C (within B) INT vs. INT+R	
Level of the other factor	B: NaCl	B: NE	A: N	A: H	C: INT	C: INT+R	A: N	A: H	C: INT	C: INT+R	B: NaCl	B: NE
LV edP	0.688	0.002	0.342	0.041	<0.001	0.96	0.772	0.001	0.758			
SW	0.354				0.011	0.135	0.876	<0.001	0.08			
SV	0.505				0.03	0.419	0.301	<0.001	<0.001	0.597	0.32	<0.001
HR	0.544				0.012	0.38	0.048	0.186	0.056	0.019	0.065	0.015
CI	0.668				0.01	0.477	0.828	<0.001	0.170			
LV dP/dt min	0.221				0.01	0.004	0.37	<0.001	<0.001	0.355	0.088	<0.001
EF	0.461				0.037	0.263	0.348	<0.001	0.226			

The table shows the *p* values for the interactions between the factors. ----- Significance not calculated because it is included in the three-factor interaction. For LV edV, RVSP, RV dP/dt max, and RV dP/dt min, there were no significant interactions.

**Table 4:** Hemodynamic parameters.

		NaCl				NE		
	N-NaCl	H-NaCl	N-NaCl+R	H-NaCl+R	N-NE	H-NE	N-NE+R	H-NE+R
LV dP/dt min (mmHg/s)	−11896 ± 468	−10175 ## ± 614	−10570 ± 1091	−14055 •• ± 344	−8464 ** ± 633	−6668 *** ± 461	−11069 ± 820	−12126 ••• ± 854
RV dP/dt max (mmHg/s)	2307 ± 183	2458 ± 192	2456 ± 274	2583 ± 226	2448 ± 158	2686 ± 190	2445 ± 151	2521 ± 141
RV dP/dt min (mmHg/s)	−2098 ± 174	−1928 ± 137	−1986 ± 195	−2348 ± 167	−2024 ± 144	−2148 ± 151	−1996 ± 117	−2262 ± 152
EF (%)	61.8 ± 1.2	54.1 ± 2.7	64.4 ± 4.0	64.9 ± 6.1	49.8 ± 3.1	41.8 * ± 6.0	54.8 ± 5.9	64.3 • ± 2.1
LV edV (μL)	311.0 ± 9.8	279.6 ± 9.7	274.6 ± 21.3	271.8 ± 14.5	282.3 ± 10.1	275.5 ± 9.4	266.2 ± 14.1	299.8 ± 9.3
DAP (mmHg)	98.4 ± 3.9	79.8 ** # ± 3.4	80.9 • ± 5.7	100.0 •• ° ± 2.7	82.7 * # ± 4.0	63.6 *** ± 2.8	103.4 •• °° ± 3.4	95.1 ••• ± 2.5
MAP (mmHg)	109.6 ± 3.5	91.4 ** ± 3.6	91.4 • ± 5.6	111.9 •• ° ± 3.2	94.7 # ± 4.0	77.9 *** ± 3.0	117.2 ••• °°° ± 3.5	108.0 ••• ± 2.9
TPR (mmHg min kg mL^−1^)	0.29 ± 0.01	0.27 ± 0.02	0.24 ± 0.02	0.30 ± 0.02	0.33 ± 0.02	0.42 ± 0.08	0.41 ° ± 0.05	0.31 ± 0.01

LV and RV maximum rate of pressure decrease (LV dP/dt min, RV dP/dt min); RV maximum rate of pressure increase (RV dP/dt max); ejection fraction (EF); LV end-diastolic pressure (LV edP); diastolic aortic pressure (DAP); mean aortic pressure (MAP); total peripheral resistance (TPR). Data are presented as mean ± SEM. Significance marks: significant differences vs. N-NaCl: * *p* < 0.05; ** *p* < 0.01; *** *p* < 0.001; vs. H-NE: # *p* < 0.05; ## *p* < 0.01; vs. N-NaCl+R: ° *p* < 0.05; °° *p* < 0.01; °°° *p* < 0.001; significant differences vs. corresponding intervention period: • *p* < 0.05; •• *p* < 0.01; ••• *p* < 0.001.

## Data Availability

The original contributions presented in this study are included in the article. Further inquiries can be directed to the corresponding author.

## Abbreviations

The following abbreviations are used in this manuscript:

ADP	adenosine diphosphate
AIF	apoptosis-inducing factor
AMP	adenosine monophosphate
ANOVA	analysis of variance
ATP	adenosine triphosphate
BW	body weight
c	concentration
cHb	concentration of hemoglobin
CI	cardiac index
DAP	diastolic aortic pressure
dP/dt max	maximum rate of pressure increase
dP/dt min	maximum rate of pressure decrease
EF	ejection fraction
H	normobaric hypoxia (10% O_2_ in N_2_)
Hct	hematocrit
HIF	hypoxia-inducible factor
HR	heart rate
I/R	ischemia/reperfusion
LV	left cardiac ventricle
LV edP	left ventricular end-diastolic pressure
LV edV	left ventricular end-diastolic volume
LVSP	left ventricular systolic peak pressure
MAP	mean aortic pressure
N	normoxia
NaCl	0.9% sodium chloride solution
NE	norepinephrine
NO	nitric oxide
NT	nitrotyrosine
PAR	poly(ADP-ribose)
PARP-1	PAR polymerase-1
pCO_2_	partial pressure of carbon dioxide
pO_2_	partial pressure of oxygen
+R	plus recovery period
RNS	reactive nitrogen species
ROS	reactive oxygen species
RV	right cardiac ventricle
RVSP	right ventricular systolic peak pressure
SaO_2_	arterial saturation of oxygen
SEM	standard error of the mean
SV	stroke volume
SW	stroke work
TPR	total peripheral resistance

## References

[1] Neubert E., Rassler B., Hoschke A., Raffort C., Salameh A. (2023). Effects of Normobaric Hypoxia and Adrenergic Blockade over 72 h on Cardiac Function in Rats. Int. J. Mol. Sci..

[2] Bölter C., Gabriel P., Appelt P., Salameh A., Schierle K., Rassler B. (2019). Effects of Adrenergic Agonists and Antagonists on Cardiopulmonary Function During Normobaric Hypoxia in Rat. Front. Physiol..

[3] Bambor C., Daunheimer S., Raffort C., Koedel J., Salameh A., Rassler B. (2025). Effects of a three-day vs. six-day exposure to normobaric hypoxia on the cardiopulmonary function of rats. Curr. Issues Mol. Biol..

[4] Talbot N.P., Balanos G.M., Dorrington K.L., Robbins P.A. (2005). Two temporal components within the human pulmonary vascular response to approximately 2 h of isocapnic hypoxia. J. Appl. Physiol..

[5] Yan B., Hu Y., Ji H., Bao D. (2007). The effect of acute hypoxia on left ventricular function during exercise. Eur. J. Appl. Physiol..

[6] Maufrais C., Rupp T., Bouzat P., Doucende G., Verges S., Nottin S., Walther G. (2017). Heart mechanics at high altitude: 6 days on the top of Europe. Eur. Heart J. Cardiovasc. Imaging.

[7] Stembridge M., Ainslie P.N., Hughes M.G., Stöhr E.J., Cotter J.D., Nio A.Q., Shave R. (2014). Ventricular structure, function, and mechanics at high altitude: Chronic remodeling in Sherpa vs. short-term lowlander adaptation. J. Appl. Physiol..

[8] Osculati G., Revera M., Branzi G., Faini A., Malfatto G., Bilo G., Giuliano A., Gregorini F., Ciambellotti F., Lombardi C. (2016). Effects of hypobaric hypoxia exposure at high altitude on left ventricular twist in healthy subjects: Data from HIGHCARE study on Mount Everest. Eur. Heart J. Cardiovasc. Imaging.

[9] Leuenberger U., Gleeson K., Wroblewski K., Prophet S., Zelis R., Zwillich C., Sinoway L. (1991). Norepinephrine clearance is increased during acute hypoxemia in humans. Am. J. Physiol..

[10] Bärtsch P., Gibbs J.S. (2007). Effect of altitude on the heart and the lungs. Circulation.

[11] Dedobbeleer C., Hadefi A., Naeije R., Unger P. (2013). Left ventricular adaptation to acute hypoxia: A speckle-tracking echocardiography study. J. Am. Soc. Echocardiogr..

[12] Rao M., Li J., Qin J., Zhang J., Gao X., Yu S., Yu J., Chen G., Xu B., Li H. (2015). Left ventricular function during acute high-altitude exposure in a large group of healthy young Chinese men. PLoS ONE.

[13] Saito M., Mano T., Iwase S., Koga K., Abe H., Yamazaki Y. (1988). Responses in muscle sympathetic activity to acute hypoxia in humans. J. Appl. Physiol..

[14] Hansen J., Sander M. (2003). Sympathetic neural overactivity in healthy humans after prolonged exposure to hypobaric hypoxia. J. Physiol..

[15] Kacimi R., Richalet J.P., Corsin A., Abousahl I., Crozatier B. (1992). Hypoxia-induced downregulation of beta-adrenergic receptors in rat heart. J. Appl. Physiol..

[16] Essop M.F., Razeghi P., McLeod C., Young M.E., Taegtmeyer H., Sack M.N. (2004). Hypoxia-induced decrease of UCP3 gene expression in rat heart parallels metabolic gene switching but fails to affect mitochondrial respiratory coupling. Biochem. Biophys. Res. Commun..

[17] Heather L.C., Cole M.A., Tan J.J., Ambrose L.J., Pope S., Abd-Jamil A.H., Carter E.E., Dodd M.S., Yeoh K.K., Schofield C.J. (2012). Metabolic adaptation to chronic hypoxia in cardiac mitochondria. Basic Res. Cardiol..

[18] Kierans S.J., Taylor C.T. (2021). Regulation of glycolysis by the hypoxia-inducible factor (HIF): Implications for cellular physiology. J. Physiol..

[19] Hernansanz-Agustín P., Enríquez J.A. (2021). Generation of Reactive Oxygen Species by Mitochondria. Antioxidants.

[20] Dwyer K.D., Snyder C.A., Coulombe K.L.K. (2025). Cardiomyocytes in Hypoxia: Cellular Responses and Implications for Cell-Based Cardiac Regenerative Therapies. Bioengineering.

[21] Görlach A., Bertram K., Hudecova S., Krizanova O. (2015). Calcium and ROS: A mutual interplay. Redox Biol..

[22] Dridi H., Santulli G., Bahlouli L., Miotto M.C., Weninger G., Marks A.R. (2023). Mitochondrial Calcium Overload Plays a Causal Role in Oxidative Stress in the Failing Heart. Biomolecules.

[23] Jung F., Palmer L.A., Zhou N., Johns R.A. (2000). Hypoxic regulation of inducible nitric oxide synthase via hypoxia inducible factor-1 in cardiac myocytes. Circ. Res..

[24] Alvarez M.N., Trujillo M., Radi R. (2002). Peroxynitrite formation from biochemical and cellular fluxes of nitric oxide and superoxide. Methods Enzymol..

[25] Radi R. (2013). Peroxynitrite, a stealthy biological oxidant. J. Biol. Chem..

[26] Szabó C., Ischiropoulos H., Radi R. (2007). Peroxynitrite: Biochemistry, pathophysiology and development of therapeutics. Nat. Rev. Drug Discov..

[27] Islam B.U., Habib S., Ali S.A., Moinuddin, Ali A. (2017). Role of Peroxynitrite-Induced Activation of Poly(ADP-Ribose) Polymerase (PARP) in Circulatory Shock and Related Pathological Conditions. Cardiovasc. Toxicol..

[28] Wang Y., Dawson V.L., Dawson T.M. (2009). Poly(ADP-ribose) signals to mitochondrial AIF: A key event in parthanatos. Exp. Neurol..

[29] David K.K., Andrabi S.A., Dawson T.M., Dawson V.L. (2009). Parthanatos, a messenger of death. Front. Biosci. (Landmark Ed.).

[30] Bárány T., Simon A., Szabó G., Benkő R., Mezei Z., Molnár L., Becker D., Merkely B., Zima E., Horváth E.M. (2017). Oxidative Stress-Related Parthanatos of Circulating Mononuclear Leukocytes in Heart Failure. Oxid. Med. Cell. Longev..

[31] Fang Q., Li Y., Wang Y., Mu N., Ma H., Yu L. (2025). Parthanatos: A redox-dependent cell death pathway in cardiovascular disease and myocardial aging. Pathol. Res. Pract..

[32] Pacher P., Szabó C. (2007). Role of poly(ADP-ribose) polymerase 1 (PARP-1) in cardiovascular diseases: The therapeutic potential of PARP inhibitors. Cardiovasc. Drug Rev..

[33] Singh M., Thomas P., Shukla D., Tulsawani R., Saxena S., Bansal A. (2013). Effect of subchronic hypobaric hypoxia on oxidative stress in rat heart. Appl. Biochem. Biotechnol..

[34] Aguilar M., González-Candia A., Rodríguez J., Carrasco-Pozo C., Cañas D., García-Herrera C., Herrera E.A., Castillo R.L. (2018). Mechanisms of Cardiovascular Protection Associated with Intermittent Hypobaric Hypoxia Exposure in a Rat Model: Role of Oxidative Stress. Int. J. Mol. Sci..

[35] Fu Y.C., Yin S.C., Chi C.S., Hwang B., Hsu S.L. (2006). Norepinephrine induces apoptosis in neonatal rat endothelial cells via a ROS-dependent JNK activation pathway. Apoptosis.

[36] Corbi G., Conti V., Russomanno G., Longobardi G., Furgi G., Filippelli A., Ferrara N. (2013). Adrenergic signaling and oxidative stress: A role for sirtuins?. Front. Physiol..

[37] Thakur A., Alam M.J., Ajayakumar M.R., Ghaskadbi S., Sharma M., Goswami S.K. (2015). Norepinephrine-induced apoptotic and hypertrophic responses in H9c2 cardiac myoblasts are characterized by different repertoire of reactive oxygen species generation. Redox Biol..

[38] Waller C., Rhee D.S., Gröger M., Rappel M., Maier T., Müller M., Rottler E., Nerz K., Nerz C., Brill S. (2020). Social Stress-Induced Oxidative DNA Damage Is Related to Prospective Cardiovascular Risk. J. Clin. Med..

[39] Kim G.T., Chun Y.S., Park J.W., Kim M.S. (2003). Role of apoptosis-inducing factor in myocardial cell death by ischemia-reperfusion. Biochem. Biophys. Res. Commun..

[40] Dostar Y., Gorjani A., Hashemi M., Shahir R.R. (2017). The effect of time on apoptosis changes following ischemia-reperfusion in isolated heart of rats. Asia Pac. J. Cancer Biol..

[41] Zenebe W.J., Nazarewicz R.R., Parihar M.S., Ghafourifar P. (2007). Hypoxia/reoxygenation of isolated rat heart mitochondria causes cytochrome c release and oxidative stress; evidence for involvement of mitochondrial nitric oxide synthase. J. Mol. Cell. Cardiol..

[42] Irlbeck M., Mühling O., Iwai T., Zimmer H.-G. (1996). Different response of the rat left and right heart to norepinephrine. Cardiovasc. Res..

[43] Barth W., Deten A., Bauer M., Reinohs M., Leicht M., Zimmer H.-G. (2000). Differential Remodeling of the Left and Right Heart After Norepinephrine Treatment in Rats: Studies on Cytokines and Collagen. J. Mol. Cell. Cardiol..

[44] Rassler B., Reissig C., Briest W., Tannapfel A., Zimmer H.G. (2003). Catecholamine-induced pulmonary edema and pleural effusion in rats—alpha- and beta-adrenergic effects. Respir. Physiol. Neurobiol..

[45] Abramoff M.D., Magalhaes P.J., Ram S.J. (2004). Image processing with ImageJ. Biophotonics Int..

[46] Reddan B., Cummins E.P. (2025). The regulation of cell metabolism by hypoxia and hypercapnia. J. Biol. Chem..

[47] Brun-Pascaud M., Gaudebout C., Blayo M.C., Pocidalo J.J. (1982). Arterial blood gases and acid-base status in awake rats. Respir. Physiol..

[48] Dempsey J.A., Powell F.L., Bisgard G.E., Blain G.M., Poulin M.J., Smith C.A. (2014). Role of chemoreception in cardiorespiratory acclimatization to, and deacclimatization from, hypoxia. J. Appl. Physiol..

[49] Dempsey J.A., Forster H.V., Bisgard G.E., Chosy L.W., Hanson P.G., Kiorpes A.L., Pelligrino D.A. (1979). Role of cerebrospinal fluid [H+] in ventilatory deacclimatization from chronic hypoxia. J. Clin. Investig..

[50] Wasse L.K., Sunderland C., King J.A., Batterham R.L., Stensel D.J. (2012). Influence of rest and exercise at a simulated altitude of 4000 m on appetite, energy intake, and plasma concentrations of acylated ghrelin and peptide YY. J. Appl. Physiol..

[51] Aeberli I., Erb A., Spliethoff K., Meier D., Götze O., Frühauf H., Fox M., Finlayson G.S., Gassmann M., Berneis K. (2013). Disturbed eating at high altitude: Influence of food preferences, acute mountain sickness and satiation hormones. Eur. J. Nutr..

[52] Matu J., O’Hara J., Hill N., Clarke S., Boos C., Newman C., Holdsworth D., Ispoglou T., Duckworth L., Woods D. (2017). Changes in appetite, energy intake, body composition, and circulating ghrelin constituents during an incremental trekking ascent to high altitude. Eur. J. Appl. Physiol..

[53] Dünnwald T., Gatterer H., Faulhaber M., Arvandi M., Schobersberger W. (2019). Body Composition and Body Weight Changes at Different Altitude Levels: A Systematic Review and Meta-Analysis. Front. Physiol..

[54] Honig A. (1989). Peripheral arterial chemoreceptors and reflex control of sodium and water homeostasis. Am. J. Physiol..

[55] Siebenmann C., Robach P., Lundby C. (2017). Regulation of blood volume in lowlanders exposed to high altitude. J. Appl. Physiol..

[56] Hildebrandt W., Ottenbacher A., Schuster M., Swenson E.R., Bärtsch P. (2000). Diuretic effect of hypoxia, hypocapnia, and hyperpnea in humans: Relation to hormones and O_2_ chemosensitivity. J. Appl. Physiol..

[57] Haditsch B., Roessler A., Krisper P., Frisch H., Hinghofer-Szalkay H.G., Goswami N. (2015). Volume regulation and renal function at high altitude across gender. PLoS ONE.

[58] Zubieta-Calleja G.R., Paulev P.E., Zubieta-Calleja L., Zubieta-Castillo G. (2007). Altitude adaptation through hematocrit changes. J. Physiol. Pharmacol..

[59] Watts D., Gaete D., Rodriguez D., Hoogewijs D., Rauner M., Sormendi S., Wielockx B. (2020). Hypoxia Pathway Proteins are Master Regulators of Erythropoiesis. Int. J. Mol. Sci..

[60] Holloway C., Cochlin L., Codreanu I., Bloch E., Fatemian M., Szmigielski C., Atherton H., Heather L., Francis J., Neubauer S. (2011). Normobaric hypoxia impairs human cardiac energetics. FASEB J..

[61] Kass D.A., Bronzwaer J.G., Paulus W.J. (2004). What mechanisms underlie diastolic dysfunction in heart failure?. Circ. Res..

[62] Alexander J.K., Grover R.F. (1983). Mechanism of reduced cardiac stroke volume at high altitude. Clin. Cardiol..

[63] Severi S., Cavalcanti S., Mancini E., Santoro A. (2002). Effect of electrolyte and pH changes on the sinus node pacemaking in humans. J. Electrocardiol..

[64] Bruno R.M., Ghiadoni L., Pratali L. (2016). Vascular adaptation to extreme conditions: The role of hypoxia. Artery Res..

[65] León-Velarde F., Bourin M.C., Germack R., Mohammadi K., Crozatier B., Richalet J.P. (2001). Differential alterations in cardiac adrenergic signaling in chronic hypoxia or norepinephrine infusion. Am. J. Physiol. Regul. Integr. Comp. Physiol..

[66] Gorr M.W., Sriram K., Chinn A.M., Muthusamy A., Insel P.A. (2020). Transcriptomic profiles reveal differences between the right and left ventricle in normoxia and hypoxia. Physiol. Rep..

[67] Chandler B.M., Sonnenblick E.H., Pool P.E. (1968). Mechanochemistry of cardiac muscle. 3. Effects of norepinephrine on the utilization of high-energy phosphates. Circ. Res..

[68] Foulon P., De Backer D. (2018). The hemodynamic effects of norepinephrine: Far more than an increase in blood pressure!. Ann. Transl. Med..

[69] Lyon A.R., Citro R., Schneider B., Morel O., Ghadri J.R., Templin C., Omerovic E. (2021). Pathophysiology of Takotsubo Syndrome: JACC State-of-the-Art Review. J. Am. Coll. Cardiol..

[70] Mauriello A., Giudice C.D., Vecchio G.E.D., Correra A., Maratea A.C., Grieco M., Amata A., Quagliariello V., Maurea N., Proietti R. (2025). Takotsubo Syndrome and Oxidative Stress: Physiopathological Linkage and Future Perspectives. Antioxidants.

[71] Surikow S.Y., Nguyen T.H., Stafford I., Chapman M., Chacko S., Singh K., Licari G., Raman B., Kelly D.J., Zhang Y. (2018). Nitrosative Stress as a Modulator of Inflammatory Change in a Model of Takotsubo Syndrome. JACC Basic Transl. Sci..

[72] Thorén P.N. (1977). Characteristics of left ventricular receptors with nonmedullated vagal afferents in cats. Circ. Res..

[73] Zucker I.H. (1986). Left ventricular receptors: Physiological controllers or pathological curiosities?. Basic Res. Cardiol..

[74] Arya S., Belwal S., Uniyal B., Tiwari B., Sharma P. (2020). Bezold Jarisch Reflex- New Interest, Old Phenomenon. Am. J. Intern. Med..

[75] Wang J., Ochoa M., Patel M.B., Zucker I.H., Loud A.V., Zeballos G.A., Hintze T.H. (1991). Carotid baroreceptor function in dogs with chronic norepinephrine infusion. Hypertension.

[76] Berdeaux A., Giudicelli J.F. (1987). Antihypertensive drugs and baroreceptor reflex control of heart rate and blood pressure. Fundam. Clin. Pharmacol..

[77] Larsen T.R., Kaszala K., Tan A.Y., Ellenbogen K.A., Huizar J.F. (2018). Paradoxical reflex bradycardia after epinephrine infusion for arrhythmia induction in the electrophysiology laboratory. HeartRhythm Case Rep..

[78] Biaggioni I., Shibao C.A., Diedrich A., Muldowney J.A.S., Laffer C.L., Jordan J. (2019). Blood Pressure Management in Afferent Baroreflex Failure: JACC Review Topic of the Week. J. Am. Coll. Cardiol..

[79] Lu X.Y., Barnett D.B. (1990). Differential rates of down regulation and recovery of rat myocardial beta-adrenoceptor subtypes in vivo. Eur. J. Pharmacol..

[80] Alsadder L., Hamadah A. (2025). Cardiac Ischaemia-Reperfusion Injury: Pathophysiology, Therapeutic Targets and Future Interventions. Biomedicines.

[81] Di Lisa F., Bernardi P. (2006). Mitochondria and ischemia-reperfusion injury of the heart: Fixing a hole. Cardiovasc. Res..

[82] Dhalla N.S., Elmoselhi A.B., Hata T., Makino N. (2000). Status of myocardial antioxidants in ischemia-reperfusion injury. Cardiovasc. Res..

[83] van den Tweel E.R., Nijboer C., Kavelaars A., Heijnen C.J., Groenendaal F., van Bel F. (2005). Expression of nitric oxide synthase isoforms and nitrotyrosine formation after hypoxia-ischemia in the neonatal rat brain. J. Neuroimmunol..

[84] Hirabayashi H., Takizawa S., Fukuyama N., Nakazawa H., Shinohara Y. (2000). Nitrotyrosine generation via inducible nitric oxide synthase in vascular wall in focal ischemia-reperfusion. Brain Res..

[85] Grosche A., Freeman D.E., Morton A.J., Polyak M.M., Matyjaszek S.A. (2012). Effects of ischemia and reperfusion on production of nitrotyrosine, activation of eosinophils, and apoptosis in the large colonic mucosa of horses. Am. J. Vet. Res..

[86] Takizawa S., Fukuyama N., Hirabayashi H., Nakazawa H., Shinohara Y. (1999). Dynamics of nitrotyrosine formation and decay in rat brain during focal ischemia-reperfusion. J. Cereb. Blood Flow Metab..

[87] Nag S., Picard P., Stewart D.J. (2001). Expression of nitric oxide synthases and nitrotyrosine during blood-brain barrier breakdown and repair after cold injury. Lab. Investig..

[88] Huang P., Chen G., Jin W., Mao K., Wan H., He Y. (2022). Molecular Mechanisms of Parthanatos and Its Role in Diverse Diseases. Int. J. Mol. Sci..

[89] Lui J.C., Kong S.K. (2007). Heat shock protein 70 inhibits the nuclear import of apoptosis-inducing factor to avoid DNA fragmentation in TF-1 cells during erythropoiesis. FEBS Lett..

[90] Choudhury S., Bae S., Ke Q., Lee J.Y., Kim J., Kang P.M. (2011). Mitochondria to nucleus translocation of AIF in mice lacking Hsp70 during ischemia/reperfusion. Basic Res. Cardiol..

[91] Yang S., Zhao X., Xu H., Chen F., Xu Y., Li Z., Sanchis D., Jin L., Zhang Y., Ye J. (2017). AKT2 Blocks Nucleus Translocation of Apoptosis-Inducing Factor (AIF) and Endonuclease G (EndoG) While Promoting Caspase Activation during Cardiac Ischemia. Int. J. Mol. Sci..

[92] Zhang Y., Zhang X., Park T.S., Gidday J.M. (2005). Cerebral endothelial cell apoptosis after ischemia-reperfusion: Role of PARP activation and AIF translocation. J. Cereb. Blood Flow Metab..

[93] Sevrioukova I.F. (2011). Apoptosis-inducing factor: Structure, function, and redox regulation. Antioxid. Redox Signal..

[94] Wickman G., Julian L., Olson M.F. (2012). How apoptotic cells aid in the removal of their own cold dead bodies. Cell Death Differ..

[95] Neri M., Cerretani D., Fiaschi A.I., Laghi P.F., Lazzerini P.E., Maffione A.B., Micheli L., Bruni G., Nencini C., Giorgi G. (2007). Correlation between cardiac oxidative stress and myocardial pathology due to acute and chronic norepinephrine administration in rats. J. Cell. Mol. Med..

[96] Deo S.H., Jenkins N.T., Padilla J., Parrish A.R., Fadel P.J. (2013). Norepinephrine increases NADPH oxidase-derived superoxide in human peripheral blood mononuclear cells via α-adrenergic receptors. Am. J. Physiol. Regul. Integr. Comp. Physiol..

[97] Jiang J.P., Downing S.E. (1990). Catecholamine cardiomyopathy: Review and analysis of pathogenetic mechanisms. Yale J. Biol. Med..

[98] Cross H.R., Murphy E., Steenbergen C. (2002). Ca^2+^ loading and adrenergic stimulation reveal male/female differences in susceptibility to ischemia-reperfusion injury. Am. J. Physiol. Heart Circ. Physiol..

[99] Zhu W.Z., Wang S.Q., Chakir K., Yang D., Zhang T., Brown J.H., Devic E., Kobilka B.K., Cheng H., Xiao R.P. (2003). Linkage of beta1-adrenergic stimulation to apoptotic heart cell death through protein kinase A-independent activation of Ca2+/calmodulin kinase II. J. Clin. Investig..

[100] de Lima-Seolin B.G., Nemec-Bakk A., Forsyth H., Kirk S., da Rosa Araujo A.S., Schenkel P.C., Belló-Klein A., Khaper N. (2019). Bucindolol Modulates Cardiac Remodeling by Attenuating Oxidative Stress in H9c2 Cardiac Cells Exposed to Norepinephrine. Oxid. Med. Cell. Longev..

[101] Arnoult D., Parone P., Martinou J.C., Antonsson B., Estaquier J., Ameisen J.C. (2002). Mitochondrial release of apoptosis-inducing factor occurs downstream of cytochrome c release in response to several proapoptotic stimuli. J. Cell. Biol..

[102] Candé C., Vahsen N., Garrido C., Kroemer G. (2004). Apoptosis-inducing factor (AIF): Caspase-independent after all. Cell Death Differ..

[103] Fu Y.C., Chi C.S., Yin S.C., Hwang B., Chiu Y.T., Hsu S.L. (2004). Norepinephrine induces apoptosis in neonatal rat cardiomyocytes through a reactive oxygen species-TNF alpha-caspase signaling pathway. Cardiovasc. Res..

[104] Lai K.B., Sanderson J.E., Yu C.M. (2009). High dose norepinephrine-induced apoptosis in cultured rat cardiac fibroblast. Int. J. Cardiol..

[105] Jain A., Atale N., Kohli S., Bhattacharya S., Sharma M., Rani V. (2015). An assessment of norepinephrine mediated hypertrophy to apoptosis transition in cardiac cells: A signal for cell death. Chem. Biol. Interact..

[106] Camargo L.L., Rios F.J., Montezano A.C., Touyz R.M. (2025). Reactive oxygen species in hypertension. Nat. Rev. Cardiol..

[107] Mortola J.P., Saiki C. (1996). Ventilatory response to hypoxia in rats: Gender differences. Respir. Physiol..

[108] St. Pierre S.R., Peirlinck M., Kuhl E. (2022). Sex Matters: A Comprehensive Comparison of Female and Male Hearts. Front. Physiol..

[109] Walker C.J., Schroeder M.E., Aguado B.A., Anseth K.S., Leinwand L.A. (2021). Matters of the heart: Cellular sex differences. J. Mol. Cell. Cardiol..

[110] Wearing O.H., Scott G.R. (2022). Sex-specific effects of chronic hypoxia on routine cardiovascular function and metabolism in CD-1 mice. Am. J. Physiol. Regul. Integr. Comp. Physiol..

[111] Lim C.C., Bryan N.S., Jain M., Garcia-Saura M.F., Fernandez B.O., Sawyer D.B., Handy D.E., Loscalzo J., Feelisch M., Liao R. (2009). Glutathione peroxidase deficiency exacerbates ischemia-reperfusion injury in male but not female myocardium: Insights into antioxidant compensatory mechanisms. Am. J. Physiol. Heart Circ. Physiol..

[112] Kander M.C., Cui Y., Liu Z. (2017). Gender difference in oxidative stress: A new look at the mechanisms for cardiovascular diseases. J. Cell. Mol. Med..

[113] Oliveira A.L.M.B., Rodrigues G.D., Silva B.M., Rohan P.A., Soares P.P.S. (2025). Sex differences in cardiorespiratory control under hypoxia: The roles of oxygen desaturation and hypoxic exposure time. Front. Cardiovasc. Med..

